# Oral manifestations in chikungunya patients: A systematic review

**DOI:** 10.1371/journal.pntd.0009401

**Published:** 2021-06-10

**Authors:** Daniela Brostolin da Costa, Alessandro Diogo De-Carli, Livia Fernandes Probst, Antonio José Grande, Ana Tereza Gomes Guerrero

**Affiliations:** 1 Postgraduate Program in Family Health/Professional Master in Family Health, Federal University of Mato Grosso do Sul, Campo Grande, Mato Grosso do Sul, Brazil; 2 Faculty of Dentistry, Federal University of Mato Grosso do Sul, Campo Grande, Mato Grosso do Sul, Brazil; 3 Piracicaba Dental School, State University of Campinas, Piracicaba, São Paulo, Brazil; 4 Medical Course, State University of Mato Grosso do Sul, Campo Grande, Mato Grosso do Sul, Brazil; 5 Institute of Technology in Immunobiologicals, Bio-Manguinhos, Oswaldo Cruz Foundation, Rio de Janeiro, Rio de Janeiro, Brazil; Australian Red Cross Lifelood, AUSTRALIA

## Abstract

**Background:**

Chikungunya fever is considered an abrupt onset arbovirus transmitted by mosquitoes, mainly *Aedes aegypti* and *Aedes albopictus*. The disease has a significant impact on the quality of life of affected persons, and many of its numerous symptoms have not yet been properly clarified, such as the manifestations that can occur in the oral cavity. The aim of this study was to identify the main oral manifestations related to chikungunya fever, as well as describe the demographic characteristics of patients, by conducting a systematic review of the literature.

**Methods and findings:**

Searches were performed in MEDLINE (PubMed), Embase (Elsevier), LILACS (VHL), Cochrane Library, Scopus, and CAPES electronic databases for theses and dissertations published up to January 16, 2021 without language and date restrictions. Additional manual searches of gray literature, reference list, and Google Scholar were carried out. We included 27 studies highlighting mainly oral manifestations that cause masticatory discomfort such as ulcers and oral thrush, gingival bleeding, pain and burning of the oral mucous membranes, temporomandibular joint (TMJ) arthralgia, opportunistic infections, and changes in taste.

**Conclusions:**

There seems to be a predominance of oral manifestations that cause discomfort when chewing, such as ulcerations in the acute phase of the disease, with complete remission within 3 to 10 days after the onset, apparently mostly affecting women and older persons. These oral manifestations can be compatible with basic viral infections related to inflammatory response and transitory immunosuppression.

## Author summary

Since its resurgence in 2004, control of the transmission of chikungunya fever has become a challenge to public health worldwide. As it is capable of diminishing the quality of life of infected persons, it has aroused interest in the scientific community in developing more in-depth studies. Many of its numerous symptoms have not yet been properly clarified, such as the manifestations that can occur in the oral cavity.

A study conducted in India in 2010, with the aim of verifying the prevalence of oral manifestations in chikungunya patients, found a positive outcome in 96.37% of the 110 suspected cases and 100% of the 37 confirmed cases.

Given the above findings, our aim in this study was to carry out a systematic review on the subject of oral manifestations in patients with chikungunya. Based on the 27 studies selected, we were able to point out the main oral manifestations that cause masticatory discomfort such as ulcers and oral thrush, gingival bleeding, pain and burning of the oral mucous membranes, temporomandibular joint (TMJ) arthralgia, opportunistic infections, and changes in taste.

Studies have reported that both frequency and worsening of oral mucosal lesions occur with increasing age and higher predominance in females. However, many studies have pointed out that most oral manifestations such as ulcerations were more frequently observed in the acute phase of the disease, with complete remission occurring 3 to 10 days after the onset of the symptoms.

## Introduction

Chikungunya fever is considered an abrupt onset arbovirus, transmitted by mosquitoes, and *Aedes aegypti* and *Aedes albopictus* are the main intermediate hosts of chikungunya virus (CHIKV). Since its resurgence in 2004, there is risk of CHIKV being imported into new areas due to the high levels of viremia in infected humans and the worldwide distribution of CHIKV transmission vectors [1].

CHIKV infection can cause acute (3 to 15 days), subacute (15 to 90 days), and chronic (>90 days) disease. When symptoms persist, they manifest mainly in the form of constant polyarthritis or polyarthralgia, accompanied by morning stiffness and asthenia, lasting from months to longer than 3 years from the onset of infection [2].

In addition to the main symptoms, studies have indicated that CHIKV fever can cause atypical manifestations, such as mucocutaneous lesions in the oral cavity, both in the acute and chronic stages of the disease [3]. Studies conducted in India during the chikungunya epidemic in 2010 showed a prevalence of 96.3% of oral manifestations, which may be located on the lips, tongue, floor of the mouth, and palate. The most frequent clinical manifestations were mucosal burning (96.3%) preceded by erythema (93.6%) and ulcers disseminated throughout the oral cavity (71.8%) [4].

Other oral manifestations were also reported such as macular depigmentation of the lips, lesions with lip crusting and lip commissures, oral mucosa pigmentation, thrush on the tongue, palate, and other areas of the oral mucosa, candidiasis on the tongue and palate, gingival pain, gingivitis, gingival bleeding, and temporomandibular joint (TMJ) arthralgia [3–5].

Although oral manifestations found in patients affected by chikungunya are similar to the pattern found in other viral infections, according to a study conducted in India in 2010, the presence of the clinical conditions of TMJ arthralgia, dysphagia, and ulcerations can be considered markers for chikungunya [4]. Patients with atypical mucocutaneous manifestations in the oral cavity should be referred for evaluation, treatment, and monitored for signs and symptoms.

In view of the foregoing, the aim of this study was to identify the main oral manifestations related to chikungunya fever, as well as describe the demographic characteristics of patients, by conducting a systematic review of the literature.

## Methods

### Protocol and registration

The protocol was registered at the International Prospective Register of Systematic Reviews (PROSPERO) protocol #CRD42018114631. This study was conducted according to the guidelines of Preferred Reporting Items for Systematic Reviews and Meta-Analyses (PRISMA) [6] in order to answer the following research question: “What are the oral manifestations described in chikungunya fever?”

### Eligibility criteria

We established the eligibility criteria according to the acronym PECOS (Population, Exposure, Comparator, Outcomes, and Study design). Details are described in Table 1.

**Table 1:** Characteristics of studies used for determining the eligibility criteria.

Population	Exposure	Comparator	Outcomes	Study design
Confirmed cases or suspected cases diagnosis for chikungunya	Oral manifestations in patients with CHIKV infection	No comparator has been established	Oral manifestations	Observational studies: case–control, cohort studies, or cross-sectional designs

CHIKV, chikungunya virus.

With regard to the diagnosis of chikungunya, we can consider 2 different situations:

1. Confirmed cases: suspected cases with clinical and laboratory confirmation of the disease [7].
2. Suspected cases: patients who meet the clinical criteria and/or epidemiological criteria [7].

The clinical criterion establishes that patients must present fever >38.5°C with abrupt onset and severe acute onset arthralgia/arthritis not explained by other medical conditions. In turn, the epidemiological criterion stipulates individual resides or has visited an endemic or epidemic area within 15 days before the onset of symptoms or has an epidemiological link with a confirmed case. As a great deal of research has been carried out on epidemic peaks in chikungunya, this criterion is in accordance with the Brazilian Society of Rheumatology, which establishes that in epidemic situations, patients in the acute phase may have a diagnosis established based only on clinical–epidemiological criteria, without confirmatory serology or associated hematology [7].

### Information sources and searches

The searches were performed up to January 16, 2021 in the electronic databases of MEDLINE (PubMed), Cochrane Library, Embase (Elsevier), LILACS (BVS), Scopus, and CAPES for dissertation and theses, databases without language and date restrictions. Additional manual searches were conducted in Google Scholar, gray literature, conference summaries, and reference list of articles. Supporting information (S1 Table) described in detail the databases, search dates, search strategies used and the number of studies retrieved, so the process may be repeated and updated if necessary.

### Study selection

Two review authors (DBC and ATGG) independently screened titles and abstracts and full texts for confirmation of eligibility. Disagreements were resolved by consensus, and, when necessary, by consulting a third reviewer (AJG).

### Data collection process and data items

Two review authors (DBC and ATGG) independently extracted data from the studies included using a standard data extraction form (S2 Table). On this form, we recorded the information extracted relative to country and years of publication of the study, study design, participants, diagnosis and data extraction type, oral manifestations described, and comments.

### Risk of bias in individual studies

The risk of bias in the studies included was assessed according to the Newcastle–Ottawa Scale (NOS) tool [8]. The tool is validated for case–control, cohort studies; it can be adapted to cross-sectional studies that were assessed for selection, comparability, and exposure categories for each study. The tool uses a method adding stars, with the highest-quality studies scoring up to 9 stars. The risk assessment process was performed by 2 independent reviewers (DBC and ATGG). Disagreements were resolved by consensus, and, when necessary, by consulting a third reviewer (AJG). All studies scored between 5 and 7 stars. The full assessment of the final risk of bias is reported in the Supporting information (S3 and S4 Tables).

### Data synthesis and statistical analysis

We synthesized all data descriptively in tables, as it was not possible to conduct meta-analysis due to different study designs, different age ranges, and times of measurement of oral manifestations. We used Mendeley Reference Manager to manage and store all the data extracted and Microsoft Excel to organize the tables.

## Results

### Study selection

A total of 795 studies were retrieved from the database searches. Moreover, 140 articles were excluded as duplicates, leaving 655 references that were then taken to the Rayyan QCRI application [9], a tool used for the screening process of titles and abstracts. These 655 references were screened by reading titles and abstracts, of which 598 were excluded because they did not meet the eligibility criteria. Manual searches in the reference lists of studies included and Google Scholar searches resulted in 11 articles that were added.

A total of 68 full texts were evaluated to confirm eligibility, and of these, 41 were excluded. The full reference list of each study excluded and reasons for exclusion are detailed in the Supporting information (S5 Table). In total, 27 studies were included. The study selection process is described in the Supporting information (S1 Fig).

### Study characteristics

The 27 articles selected were on studies conducted in India [4,10–21], Reunion Island [22–25], Sri Lanka [26], Germany [27], France [28], Colombia [29], Grenada [30], Bangladesh [31], Brazil [32–33], Pakistan [34], and Nicaragua [35], published from 2007 to 2020.

Initially, we present the characterization of all 27 studies included in Table 2.

**Table 2:** Characteristics of the studies included.

**First author (year) [ref]**	**Country and year of study**	**Study design**	***N* total gender: M/F**	**Age**	**Diagnosis type/date extraction type**	**Oral manifestations**	**Comments**
Anshul et al. (2020) [10]	India (August to November 2016)	Prospective observational cohort study	*N* = 150 59 M 91 F	1 month to 85 years	150 suspected chikungunya Only clinical examination	- 9 cases (6%) of aphthous ulcer - 9 cases (6%) of angular cheilitis	Mucosal lesions lasted for 7 to 10 days and subsided completely without any sequelae.
Bandyopadhyay et al. (2008) [11]	India (2007)	Observational historical cohort study	*N* = 26 13 M 13 F	10 to 60 years	26 confirmed chikungunya Clinical, laboratorial examinations, and self-reported (anamnesis)	- 1 case (3.8%) of oral ulcers - 1 case (3.8%) lips herpes	Contrary to other studies that showed high prevalence of oral mucosa aphthous ulcers, in the present study, the author pointed out that this type of outcome was observed in only 1 case.
Bhat et al. (2011) [12]	India (June to August 2008)	Observational historical cohort study	*N* = 75 37 M 38 F	1 to 70 years	75 suspected chikungunya Clinical, laboratorial examinations, and self-reported (anamnesis)	- 12 cases (16%) of oral ulcers	Oral ulcers were observed in both sexes, predominantly in the acute phase of the disease (2 to 7 days), presenting as multiple small ulcers (2 to 5 mm) with inflammatory aspect, painful, and covered by plaque.
Borgherini et al. (2007) [22]	Reunion Island (March 2005 to April 2006)	Observational historical cohort study	*N* = 157 87 M 70 F	16 years and over	157 confirmed chikungunya (only acute phase) Medical records and laboratorial examination	- 4 cases (2.5%) of oral ulcers - 2 cases (1.25%) of gingival bleeding	No relevant comments
Casais et al. (2020) [32]	Brazil (March 2016 to June 2017)	Prospective observational cohort study	*N* = 105 41 M 64 F	28 to 57 years	105 confirmed chikungunya Clinical and laboratory examinations	- 20 cases (19%) of dysphagia - 15 cases (14%) of oral ulcers - 7 cases (6.6%) of gingival bleeding - 3 cases (2.8%) of herpes - 1 case (1%) of gingival edema	Was detected CHIKV in saliva of 27% of patients with oral involvement suggesting that lesions may result from direct viral activity
Chang et al. (2018) [29]	Colombia (20-month segment from January 2015)	Prospective observational cohort study	*N* = 485 97 M 388 F	Mean age of 49.1 years	485 confirmed chikungunya (only chronic phase) Medical records, laboratorial examination, and self-reports (telephone survey)	- 6 cases (1.23%) of gingival bleeding	There was no statistical difference (*p* = 0.65) between joint pain and gingival bleeding significant (*p* < 0.002).
Deeba et al. (2019) [31]	Bangladesh (2017)	Cross-sectional observational study	*N* = 1,089 612 M 486 F	Aged <15 to >60	1,089 cases. Type of diagnosis not reported (only acute phase) Medical records and self-reported (questionnaire and interview)	- 414 cases (37.7%) of oral ulcers	No relevant comments
Doria (2019) [33]	Brazil (2015 to 2016)	Cross-sectional observational study	*N* = 40 4 M 36 F	20 to 68 years	40 confirmed chikungunya (only chronic phase) Clinical, laboratorial examinations, and self-reported (questionnaire)	- 4 cases (10%) of TMJ arthralgia - 3 cases (7.5%) of bitter taste in the mouth - 10 cases (25%) of oral ulcers - 8 cases (20%) of cervical lymphadenopathy - Candidiasis (number of cases not reported)	According to the prevalence of age and gender for the chronic phase of the disease, the prevalence of recurrent thrush and TMJ dysfunction was predominantly found in females and those over 45 years of age.
Fatima et al. (2020) [34]	Pakistan (July to December 2018)	Cross-sectional observational study	*N* = 531 200 M 331 F	2 to >75 years	531 confirmed chikungunya Medical records, clinical examination, and self-reported (anamnesis)	- 56 cases (10.5%) of oral ulcers - 63 cases (11.8%) of TMJ arthralgia - 261 cases (49.1%) of mouth opening pain - 100 cases (18.8%) of gingival edema - 51 cases (9.6%) of cervical lymphadenopathy	Females were affected more as compared to male gender with oral manifestation, and oral symptoms were observed more in patients 50 years old and above.
Gardner et al. (2015) [35]	Nicaragua (August 2014 to June 2015)	Prospective observational cohort study	*N* = 13 9 M 4 F	7 to 14 years	13 confirmed chikungunya (only acute phase) Medical records, clinical examination, and self-reported (anamnesis)	- 3 cases (21.4%) of gingival bleeding	This study pointed out the possibility of the presence of active CHIKV in the saliva of human patients in the acute phase of the disease.
Heath et al. (2018) [30]	Grenada (November 2015 to January 2016)	Cross-sectional observational study	*N* = 240 64 M 176 F	4 to 89 years	240 confirmed chikungunya (only chronic phase) Clinical, laboratorial examinations, and self-reported (interview)	- 87 cases (36.25%) of bitter taste in the mouth - 9 cases (3.7%) of gingival bleeding	No relevant comments
Inamadar et al. (2008) [13]	India (May to June 2006)	Prospective observational cohort study	*N* = 145 93 M 52 F	25 days to 85 years	145 suspected chikungunya Only clinical examination	- 1 case (0.68%) of tongue and palate hyperpigmentation - 1 case (0.68%) of oral ulcers - 1 case (0.68%) lip depigmentation	No relevant comments
Kannan et al. (2009) [14]	India (2007)	Cross-sectional observational study	*N* = 354 160 M 194 F	1 to 45 years	354 suspected chikungunya Medical records and self-reported (anamnesis)	- 63 cases (17.8%) of oral ulcers - 5 cases (1.4%) of gingival bleeding	No gender difference was observed for any of the symptoms. Regarding age ranges and outcomes, a higher prevalence was observed with increasing age. 1 to 15 years – *N* = 49 4 cases (8.2%) of oral ulcers 16 to 35 years – *N* = 111 17 cases (15.3%) of oral ulcers and 2 cases (1.8%) of gingival bleeding 36 to 45 years – *N* = 90 19 cases of oral ulcers and 1 case of gingival bleeding >45 years – *N* = 104 23 cases of oral ulcers and 2 cases of gingival bleeding
Katti et al. (2011) [15]	India (June to September 2006)	Prospective observational cohort study	*N* = 97 47 M 50 F	17 to 51 years	97 confirmed chikungunya Clinical, laboratorial examinations, and self-reported (anamnesis)	- 56 cases (54.32%) of gingival pain - 56 cases (54.32%) of gingival bleeding - 56 cases (54, 32%) of gingival burning - 32 cases (29.1%) of chewing difficulty - 32 cases (29.1%) of dysphagia - 22 cases (21.34%) of halitosis - 18 cases (17.46%) of oral ulcers - 12 cases (11.64%) of mouth opening pain - 6 cases (6%) of TMJ arthralgia - 10 cases (9.7%) of excess salivation - 8 cases (7.76%) of whitish plaque deposit in the gums - 1 case (0.97%) of tooth loss	Gingival pain was observed almost equally among younger individuals with 25 cases (64.1%) and older age 19 cases (67.85%). Gingival burning was more common among the younger age group with 28 cases (71.79%). Symptoms such as gingival bleeding with 26 cases (96.42%), inability to chew with 11 cases (39.28%), and halitosis with 8 cases (28.57%) were more pronounced in the age group over 45 years. Severe gingivitis was observed in patients with chronic disease, while among patients with acute disease, moderate gingivitis was observed.
Kumar et al.(2017) [16]	India (July to October 2016)	Prospective observational cohort study	*N* = 112 62 M 50 F	1 month to 77 years	76 confirmed chikungunya Clinical and laboratorial examinations	- 4 cases (3.6%) of angular cheilitis - 11 cases (9.8%) of oral ulcers - 2 cases (1.8%) of hard palate hyperpigmentation	Oral ulcers subsided within 3 to 5 days after the appearance.
Paul et al. (2011) [17]	India (June to December 2007)	Prospective observational cohort study	*N* = 100 40 M 60 F	20 to 60 years	100 confirmed chikungunya Clinical, laboratorial examinations, and self-reported (telephone interview)	- 45 cases (45%) of oral ulcers - 17 cases (17%) of glossitis	The oral ulcers were very painful and severe in some of them and appeared during the acute phase of the disease.
Razmy et al. (2014) [26]	Sri Lanka (2006)	Prospective observational cohort study	*N* = 789 318 M 470 F	<1 to >63 years	789 suspected chikungunya Only self-reports (questionnaire)	- 12 cases (1.5%) of gingival bleeding - 73 cases (9.3%) of oral ulcers (9.3%)	Patients’ gender was associated with gingival bleeding and mouth ulcers. Women presented more cases than men.
Riyaz et al. (2010) [18]	India (July to September 2009)	Prospective observational cohort study	*N* = 157 63 M 99 F	1 month to 78 years	157 confirmed chikungunya Clinical and laboratorial examinations	- 22 cases (13.64%) of oral ulcers - Cheilitis (not reported the number of cases)	Oral manifestations lasted 7 to 10 days and disappeared without sequelae.
Robin et al. (2010) [23]	Reunion Island (March 2005 to October 2006)	Observational historical cohort study	*N* = 13 8 M 5 F	Under 6 months	13 confirmed chikungunya Medical records, clinical, laboratorial examinations, and self-reported (anamnesis)	- 3 cases (23%) of oral ulcers - 1 case (7.6%) of cervical lymphadenopathy	No relevant comments
Shruti et al. (2016) [19]	India (September 2010 to August 2011)	Prospective observational cohort study	*N* = 248 Group 1 *N* = 204 131 M 73 F Group 2 *N* = 44 13 M 31 F	All age groups	Group 1 204 not confirmed chikungunya Group 2 44 confirmed chikungunya Clinical, laboratorial examinations, and self-reported (anamnesis)	Group 1 - 48 cases (23.5%) of oral ulcers Group 2 - 16 cases (36.5%) of oral ulcers	The main clinical features in the present study were fever with joint pain, rash, and aphthous ulcers on oral mucosa and tongue.
Simon et al. (2007) [28]	France (February 2005 to April 2006)	Prospective observational cohort study	*N* = 44 25 M 22 F	6 months to 73 years	44 confirmed chikungunya Clinical, laboratorial, and self-reported examinations (telephone interview and anamnesis)	- 2 cases (4.2%) of gingival bleeding - 7 cases (14.9%) of TMJ arthralgia - 1 case (2.1%) of lip herpes - 1 case (2.1%) of oral ulcers - 1 case (2.1%) of dysgeusia	No relevant comments
Singaraju et al. (2010) [4]	India (January to March 2010)	Prospective observational cohort study	Group 1 *N* = 110 47 M 63 F Group 2 *N* = 37 18 M 19 F	1 to 50 years	Group 1 110 suspected chikungunya Group 2 37 confirmed chikungunya Clinical, laboratorial examinations, and self-reported (questionnaire and anamnesis)	Group 1 - 106 cases (96.37%) of oral manifestations - 106 cases (96.37%) of oral mucosal burning - 103 cases (93.63%) of oral mucosal erythema - 79 cases (71.81%) of oral ulcers - 53 cases (48.18%) of dysphagia - 63 cases (57.27%) of TMJ arthralgia - 85 cases (77.27%) of gingivitis - 85 cases (77.27%) of cervical lymphadenopathy - 8 cases (7.27%) of interdental mucosa desquamation Group 2 - 37 cases (100%) of oral manifestations - 37 cases (100%) of oral mucosal burning - 34 cases (91.89%) of oral mucosa erythema - 32 cases (86.48%) of oral ulcers - 26 cases (70.27%) of dysphagia - 31 cases (83.78%) of TMJ arthralgia - 28 cases (75.67%) of gingivitis - 30 cases (81.08%) of cervical lymphadenopathy - 7 cases (18.91%) of interdental mucosa desquamation	Erythema-like manifestations were found on the lips, tongue, floor of the mouth. and palate. Multiple ulcers were found in the soft palate, hard palate, tongue, and floor of the mouth.
							The authors proposed the introduction of the term “Gunya stomatitis,” representing the clinical condition of TMJ arthralgia, associated with generalized erythema of the mucosa and ulcers in the oral cavity with concomitant dysphagia.
Staikowsky et al. (2009) [24]	Reunion Island (March to May 2006)	Prospective observational cohort study	*N* = 260 131 M 129 F Group A1 *N* = 180 96 M 84 F Group A2 *N* = 34 13 M 21 F Group B *N* = 46 22 M 24 F	15 to 96 years Group A1 15 to 96 years Group A2 17 to 89 years Group B 16 to 93 years	214 confirmed chikungunya Group A1 180 cases in the acute phase Group 2 Phase 34 cases in the chronic Group B 46 chikungunya not confirmed Clinical, laboratorial examinations, and self-reported (questionnaire)	Suspected Cases N = 260 -76 cases (29.4%) of dysgeusia -6 cases (2.07%) of gingival bleeding -5 cases (1.86%) of TMJ arthralgia -1 case (0.46%) of oral ulcers Group A1 -46 cases (40.4%) of dysgeusia -2 cases (1.1%) of gingival bleeding -3 cases (1.8%) of TMJ arthralgia Group A2 -17 cases (58.6%) of dysgeusia -3 cases (8.8%) of gingival bleeding -1 case (3.2%) of TMJ arthralgia -1 case (2.9% 0) of oral ulcer Group B -13 cases (35.1%) of dysgeusia -1 case (2.2%) of gingival bleeding -1 case (2.4%) of TMJ arthralgia	The study recorded rare locations of arthralgia, such as of the TMJ.
Suryawanshi et al. (2009) [20]	India (July to September 2006)	Prospective observational cohort study	*N* = 405 Group 1 *N* = 318 Gender not reported Group 2 *N* = 87 61 M 26 F	13 to 70 years	Group 1 318 suspected chikungunya Group 2 87 confirmed chikungunya Clinical, laboratorial examinations, and self-reported (anamnesis)	Group 1 -31 cases (10%) of oral ulcers -31 cases (10%) of gingivitis 4 cases (1.25%) of cervical lymphadenopathy Group 2 -9 cases (10.34%) of oral ulcers -9 cases (10.34%) of gingivitis -8 cases (9.19%) of cervical lymphadenopathy	They considered cervical lymphadenopathy and oral ulcers to be rare manifestations reported in the study.
Talarmin et al. (2007) [25]	Reunion Island (March to April 2006)	Prospective observational cohort study	*N* = 212 105 M 107 F	15 to 94 years	212 confirmed chikungunya Clinical, laboratorial examinations, and self-reported (anamnesis)	- 2 cases (0.94%) of gingival bleeding - 62 cases (29.2%) of dysgeusia - Glossitis with tongue depapillation (number of cases was not reported) - Oral mucosa erythema (number of cases was not reported) - Bitter taste (not reported the number of cases)	Mucosal involvement is frequent: pharyngitis, mouth ulcers, and glossitis with depapillation of the tongue. Dysgeusia, a bitter or metallic taste, was a good sign for diagnostic guidance.
Taubitz et al. (2007) [27]	Germany (January to October 2006)	Observational historical cohort study	*N* = 20 6 M 14 F	12 to 64 years.	20 confirmed chikungunya Clinical, laboratorial examinations, and self-reported (anamnesis)	- 1 case (5%) of gingival bleeding	No relevant comments
Vijayakumar et al. (2011) [21]	India (October to November 2007)	Cross-sectional observational study	*N* = 1,913 945 M 968 F	1 to >60 years	1,913 suspected chikungunya Self-reported (interview and questionnaire)	- 382 cases (20%) of oral ulcers	Oral ulcers occurred mainly in the first week of the disease.

CHIKV, chikungunya virus; TMJ, temporomandibular joint.

Considering the 27 studies included in this systematic review, it was possible to survey data on a total of 7,023 suspected cases of chikungunya. We could extract data from 3,854 suspected cases of chikungunya reported in 8 studies [4,10,12–14,20,21,26] and from 2,611 laboratory-confirmed cases of chikungunya reported in 18 studies [11,15–19,22–25,27–30,32–35]. A study of 1,089 participants did not report the diagnostic criteria used for determining the disease [31].

Considering that chikungunya affects all ages and both sexes, it was possible to report studies covering all age groups, and in relation to gender, 3,369 cases were studied in male participants and 4,185 cases in female patients.

In this review, we could highlight the description of the following oral manifestations identified in chikungunya fever. Among the 7,615 participants of the 27 included studies, 1,297 presented oral ulcers and thrush, the most prevalent types of manifestations reported. Among the injuries identified in over 100 patients, we observed 273 cases of mouth opening pain, 162 pain and burning of oral mucosae, 157 cervical lymphadenopathy, 148 loss of taste, 147 TMJ arthralgia, 126 dysgeusia, 123 gingivitis, 110 gingival bleeding, 105 dysphagia, 103 erythema, and 100 cases of gingival edema. Finally, the less common manifestations were cases of the following: 90 bitter taste, 32 difficulty with chewing, 22 halitosis, 17 glossitis, 13 cheilitis, 10 hypersalivation, 8 interdental mucosal desquamation, 8 herpes, and 4 cases of pigmentation of lips, tongue, and hard palate. The table showing the combined proportion of oral manifestations separated into the subgroups of suspected and confirmed cases may be found in the Supporting information (S1 Data).

### Risk of bias within studies

The assessment of the methodological quality of the studies included was conducted based on the NOS for cohort and cross-section studies, and these were assessed for selection, comparability, and outcomes. All studies were considered moderate risk of bias since they provided explanation on methodology. A total of 21 cohort studies were assessed [4,10–13,15–20,22–29,32,35] and 6 cross-sectional studies [14,21,30,31,33,34]. The scores attributed to studies ranged between 5 and 7 stars.

The bias was higher in the studies assessed with regard to intentional samples or those that were not representative of the exposed population or when clinical and methodological heterogeneity were observed across studies. This also occurred when there was lack of comparability between patients exposed and not exposed to the disease and lack of adequate control over confounding factors.

The cohort studies indicated the follow-up time and final population and how they performed the analysis of the results. In the cross-sectional studies, the statistical test used to analyze the data was clearly described and appropriate, and the measurement of association was given, including confidence intervals and the probability level (*p*-value). The analysis for each study is shown in detail in the Supporting information (S3 and S4 Tables).

## Discussion

In this review, we mapped the evidence that described oral manifestations identified in chikungunya fever. The oral manifestations inclued a miscellaneous of lesions such as oral ulcers and thrush, gingival bleeding, gingivitis, oral mucosal pain and burning, erythema, and pigmentation of the lips, tongue and hard palate, herpes, cheilitis, glossitis, interdental mucosa desquamation, dysphagia, dysgeusia, difficulty with chewing, metallic taste, TMJ arthralgia, cervical lymphadenopathy, hypersalivation and halitosis.

We observed that the frequency and worsening of oral mucosal lesions occurred with increasing age and higher predominance in females. However, many studies pointed out that the majority of oral manifestations such as ulcerations were more often observed in the acute phase of the disease, with complete remission occurring 3 to 10 days after the onset of the symptoms.

Patients with chikungunya may have several oral manifestations simultaneously and varying degrees of severity. Oral symptoms are very distressing, causing considerable pain and suffering, negatively impacting the patients’ quality of life and well-being [3]. Although it is a self-limiting disease, palliative measures to alleviate the patients’ suffering and discomfort should be used, and, as a follow-up routine, we recommend that patients should be referred to a dentist for evaluation, treatment, and monitoring of signs and symptoms.

Although oral manifestations are still considered atypical manifestations of chikungunya, the high number of records, particularly of ulcers, shows that they are much more common than is supposed. Furthermore, studies generally included the analysis of these manifestations only as a secondary outcome, a fact that made it likely that they were underestimated [3]. Most studies have not measured oral lesions or considered them an uncommon manifestation of this disease. Only 5 studies in the literature [15,20,33–35] consulted dealt exclusively with determination of the oral manifestations of this viral fever. A study conducted in India in 2010, with the aim of verifying the prevalence of oral manifestations in chikungunya patients, found a positive outcome in 96.37% of the 110 suspected cases and 100% of the 37 confirmed cases [4].

Since 1963, the Indian population has suffered from outbreaks of chikungunya. In December 2005, during a major epidemic that started with 180,000 confirmed cases, the African genotype of CHIKV was isolated in the country for the first time. In this same epidemic, it was shown that the prevalence of mucocutaneous manifestations in chikungunya patients was observed from a series of cases. These included various patterns of hyperpigmentation, vesicobullous lesions, aphthous ulcers, lesions, vasculitis, and lichenoid reaction. Hypothetically, these findings could be attributed to the entry of the African CHIKV genotype during this outbreak, in contrast with previous outbreaks caused by the Asian genotype, in which these types of manifestations had not previously been reported [13].

Ulceration and aphthous lesions were observed predominantly during the acute phase of the disease (2 to 7 days) [4,12,16–18,21] and could be isolated; they were multiple, inflammatory, painful, and small (2 to 5 mm in size) [12], with remission without sequelae in periods lasting from 3 to 10 days [16–18]. The onset of oral ulcers normally associated with erythema in the affected region was reported, and no part of the oral cavity appeared to be immune to these manifestations. Soft palate ulcers were the most common, followed by those in the hard palate, tongue, and floor of the mouth. Palatine erythema was invariably associated with multiple ulcers [4]. Identifying and treating these injuries are important to ensure that the patient is able to eat properly.

Although hemorrhagic manifestations are considered uncommon in patients with chikungunya [14], in one study with 97 confirmed chikungunya, we observed the report that 54.32% patients tested suffered from bleeding in the oral cavity, causing chewing discomfort [15]. Several studies also reported the presence of hemorrhagic manifestations in the oral cavity, especially gingival bleeding [4,14,15,20,22,24–30,35]. However, these episodes tended to be much less severe compared with those observed in dengue patients [12,16,18].

It is important to note the presence of active CHIKV in the saliva of human patients with acute infection. This evidence claims that the viral agent in saliva may be associated with hemorrhagic lesions in the nasal/oral cavities during the viremic period and points out the importance of the relationship for clinical practice and management of the patient with chikungunya by the dentist [32–35]. In a study with 105 confirmed cases for chikungunya, CHIKV was isolated in the saliva of 27% of the cases with the presence of oral manifestations [32].

Chikungunya also compromised patients’ oral hygiene status. This may be because severe gingival pain could have prevented patients from performing thorough tooth brushing. When 97 confirmed chikungunya patients were compared in terms of oral hygiene status, gingival status, and plaque accumulation during the different stages of the disease, severe gingivitis was observed in 64.7% patients with chronic disease, while mild to moderate gingivitis was found in 64.7% patients in the acute stage. However, no significant associations were observed regarding oral hygiene status and plaque scores. The difference can probably be attributed to the inability of chronic patients to clean the oral cavity properly for a longer time, which was reflected in the higher plaque scores in these patients [15].

In a systematic review of the association between oral manifestations and dengue, the hypothesis was that the occurrence of associated comorbidities and/or the previous presence of dental biofilm or calculus on the tooth surfaces may have synergistically led to the oral manifestation of dengue hemorrhagic fever in some of the reported cases [36].

A study in India, with 97 cases of confirmed chikungunya, reported 57.7% cases of severe pain and burning sensation in the oral mucosa. The description of oral mucosal pain included a burning sensation, erythema, and gingivitis that could occur from 1 to 2 days before or after the onset of clinical symptoms of the disease. The burning sensation associated with the erythema of parts or the entire oral cavity was the symptom most reported by the patients studied, with the lips, tongue, floor of the mouth, and palate being the structures most frequently involved [4].

In the majority of patients, the interdental gum was commonly affected by bulbous edema. Other nonspecific findings included interdental mucosal peeling, which may coincide with the onset of viral infection [4].

The effect of CHIKV infection on connective tissue was reported, stating that in these cases, there was increased excretion of urinary proline and hydroxyproline, which suggested a high degree of renewal in connective tissue. Whether this affected the integrity of gingival tissue and alveolar bone, causing changes in gingival and periodontal health, has not yet been confirmed. Further studies with associated radiographic examination could help determine the effects on the alveolar bone and periodontal complex in more detail [37].

Patients expressed their inability to chew varieties of food and had to limit their diet to soft, semisolid, and only liquid foods in some more severe cases. Moreover, these chewing discomforts occurred mainly during the acute phase of the disease [15] and may be closely related to cases of TMJ arthralgia, which has been found to be clinically significant in seropositive patients, with a prevalence of 83.78% [4].

Relative to the causes of dysphagia, these can be directly associated with the presence of multiple palatal erythema accompanied by ulcers and/or erythema associated with burning sensation of the oral structures. In addition, in a study with 37 confirmed chikungunya cases in India in 2010, the presence of signs and symptoms of TMJ arthralgia, dysphagia, and ulcerations was statistically significant in chikungunya seropositive cases. Thus, the authors proposed the introduction of the term “Gunya stomatitis” to represent this clinical condition [4].

Chikungunya viral fever can also induce transient immune depression, leading to the entry of potential opportunistic infections, such as candidiasis, herpes, and stomatitis (multiple ulcers), which may cause discomfort and pain during the patients’ chewing process [16,23,33]. Another clinical feature that may negatively affect masticatory function and causes dysphagia is cervical lymphadenopathy due to sublingual and submandibular lymph node edema [4,20,23,33].

The profile of chikungunya-infected patients hospitalized due to the worsening of the clinical condition consisted of older patients with a history of associated comorbidities. Chronic diseases, such as diabetes, hypertension, ischemic heart disease, lung and kidney problems, and obesity, among others, played a decisive role in the outcome of cases, prolonging the length of these hospitalizations or even causing the death of these patients [22,24,25,27,29]. Chikungunya probably played an indirect role in these fatalities, indicating that further studies of its pathogenicity are clearly needed, taking the presence of preexisting comorbidities into consideration [22].

Exacerbation or recurrent acuteness of preexisting dermatoses, such as psoriasis, lichen planus, pityriasis rosea, pityriasis alba, allergic contact dermatitis, stasis eczema, Hansen type 1, and pemphigus vulgaris, has been well documented in association with chikungunya fever [12,16,18].

There are a variety of other clinical manifestations that may be associated with chikungunya infection in addition to the well-known classical symptoms, as previously described in the literature. However, many of these symptoms are nonspecific and may be present in several other viral infections. Some of these clinical symptoms may also correspond to the decompensation of underlying conditions and iatrogenic effects such as polypharmacy, emphasizing that more specific studies on the subject are needed [24].

As limitations of the studies included, we could highlight intentional sample or nonrepresentative cases recruited from the population, increasing risk of selection bias. Furthermore, some studies used samples with suspected cases, with diagnosis established, based only on clinical–epidemiological criteria, without confirmatory serology or associated hematology.

Moreover, we point out one of the problems of assessing the cause of oral manifestations, either by virus–host interaction, drug therapy, or association with preexisting comorbidities. We were also unable to assess whether oral manifestations are prodromal symptoms or a part of the systemic involvement of viral fever. Some studies included reported oral manifestations as a secondary outcome, presenting only the percentage and number of cases of their occurrence; therefore, we were unable to perform meta-analysis or any type of statistical inference regarding the prevalence of oral manifestations.

Due to the relevance of the topic and increasing number of infections worldwide, we reiterate the need for robust studies with representative samples and comparator groups.

## Conclusions

Based on the 27 studies included, we were able to identify the main oral manifestations such as ulcers and oral thrush, gingival bleeding, pain and burning in the oral mucous membranes, arthralgia of the TMJ, opportunistic infections, and changes in taste.

Finally, we concluded that relative to oral manifestations that cause discomfort when chewing, such as ulcerations, there seems to be a predominance in the acute phase of the disease, with complete remission occurring 3 to 10 days after the onset, apparently mostly affecting women and older persons. These oral manifestations can be compatible with basic viral infections related to inflammatory response and transitory immunosuppression.

**Key learning points**

1. Chikungunya fever is considered an abrupt onset arbovirus. Since its resurgence in 2004, there is risk of chikungunya virus (CHIKV) being imported into new areas due to the high levels of viremia in infected humans and the worldwide distribution of CHIKV transmission vectors.
2. CHIKV fever can cause atypical manifestations, such as mucocutaneous lesions in the oral cavity, both in the acute and chronic stages of the disease. Frequency and worsening of oral mucosal lesions occur with increasing age and higher predominance in females.
3. Most oral manifestations such as ulcerations were more frequently observed in the acute phase of the disease, with complete remission occurring 3 to 10 days after the onset of the symptoms.
4. Oral symptoms are very distressing, causing considerable pain and suffering, negatively impacting the patients’ quality of life and well-being, and palliative measures to alleviate the patients’ suffering and discomfort should be used.
5. Patients with chikungunya should be referred to a dentist for evaluation, treatment, and monitoring of oral signs and symptoms.

**Top five papers**

1. Yanduri S. Chikungunya fever: an update for the oral health care practitioner. Int J Clin Dent Sci. 2011 May;2(2):65–68. Available from: https://core.ac.uk/download/pdf/288292422.pdf
2. Singaraju GS, Vanaja E, Sathe PS. Oral manifestations of chikungunya fever in clinically diagnosed chikungunya cases (CDCG)-a purposive study. Ann Essences Dent. 2010;2(4):25–29. doi: 10.5368/aedj.2010.2.4.25–29
3. Bhat RM, Rai Y, Ramesh A, Nandakishore B, Sukumar D, Martis J, et al. Mucocutaneous manifestations of chikungunya Fever: a study from an epidemic in coastal karnataka. Indian J Dermatol. 2011;56(3):290–294. doi: 10.4103/0019-5154.82483
4. Katti R, Shahapur PR, Udapudi KL. Impact of chikungunya virus infection on oral health status: an observational study. Indian J Dent Res. 2011;22(4):613. doi: 10.4103/0970-9290.90325
5. Kumar R, Sharma MK, Jain SK, Yadav SK, Singhal AK. Cutaneous manifestations of chikungunya fever: observations from an outbreak at a Tertiary Care Hospital in Southeast Rajasthan, India. Indian Dermatol Online J. 2017;8(5):336–342. doi: 10.4103/idoj.IDOJ_429_16

## Supporting information

S1 Table Search strategies. (DOCX)

S2 Table Data extraction. (DOCX)

S3 Table Newcastle–Ottawa risk of bias tool for cohort studies. (DOCX)

S4 Table Newcastle–Ottawa risk of bias tool for cross-sectional studies. (DOCX)

S5 Table Studies excluded with reasons for exclusion. (DOCX)

S1 Fig PRISMA flow diagram. PRISMA, Preferred Reporting Items for Systematic Reviews and Meta-Analyses.
(DOCX)

S1 Data Combined proportion of oral manifestations stratified by suspected and confirmed cases. (XLSX)

## References

[1] Staples JE, Breiman RF, Powers AM. Chikungunya fever: an epidemiological review of a re-emerging infectious disease. Clin Infect Dis. 2009;49(6):942–8. doi: 10.1086/605496 19663604

[2] Koga RdCR. Aspectos clínicos e sorológicos de indivíduos com sinais e sintomas de febre chikungunya. Goiânia: Pontifícia Universidade Católica de Goiás; 2017. Dissertação de Mestrado apresentada ao Programa de Pós-Graduação Stricto Sensu em Ciências Ambientais e Saúde, área de concentração Sociedade e Saúde Available from: http://tede2pucgoiasedubr:8080/bitstream/tede/3664/2/ROSEMARYDECARVALHOROCHAKOGA.pdf.

[3] Yanduri S. Chikungunya fever: an update for the oral health care practitioner. Int J Clin Dent Sci. 2011 May;2(2):65–68. Available from: https://core.ac.uk/download/pdf/288292422.pdf.

[4] Singaraju GS, Vanaja E, Sathe PS. Oral manifestations of chikungunya fever in clinically diagnosed chikungunya cases (CDCG)-a purposive study. Ann Essences Dent. 2010;2(4):25–9. doi: 10.5368/aedj.2010.2.4.25–29

[5] Bandyopadhyay D, Ghosh SK. Mucocutaneous manifestations of Chikungunya fever. Indian J Dermatol. 2010;55(1):64–7. doi: 10.4103/0019-5154.60356 20418982 PMC2856378

[6] Moher D, Liberati A, Tetzlaff J, Altman DG, PRISMA Group. Preferred reporting items for systematic reviews and meta-analyses: the PRISMA statement. PLoS Med. 2009;6(7):e1000097. doi: 10.1371/journal.pmed.1000097 19621072 PMC2707599

[7] Marques CDL, Duarte ALBP, Ranzolin A, Dantas AT, Cavalcanti NG, Gonçalves RSG, et al. Recommendations of the Brazilian Society of Rheumatology for diagnosis and treatment of Chikungunya fever. Part 1—Diagnosis and special situations. Rev Bras Reumatol Engl Ed. 2017;57(Suppl 2):421–437. English, Portuguese. doi: 10.1016/j.rbre.2017.05.006 28751131

[8] Wells GA, Shea B, O’Connell D, Peterson J, Welch V, Losos M, Tugwell P. New Castle-Ottawa Scale. Available from: http://www.ohri.ca/programs/clinical_epidemiology/oxford.asp.

[9] Ouzzani M, Hammady H, Fedorowicz Z, Elmagarmid A. Rayyan-a web and mobile app for systematic reviews. Syst Rev. 2016;5(1):210. doi: 10.1186/s13643-016-0384-4 27919275 PMC5139140

[10] Anshul V, Charu B, Amrita U. Dermatological Manifestations in Patients of Chikungunya Disease. Indian J Public Health Res Dev. 2020;11(04):872–6. Available from: http://medicopublication.com/index.php/ijphrd/article/view/10037/9395.

[11] Bandyopadhyay D, Ghosh SK. Mucocutaneous features of Chikungunya fever: a study from an outbreak in West Bengal, India. Int J Dermatol. 2008;47(11):1148–52. doi: 10.1111/j.1365-4632.2008.03817.x 18986446

[12] Bhat RM, Rai Y, Ramesh A, Nandakishore B, Sukumar D, Martis J, et al. Mucocutaneous manifestations of chikungunya Fever: a study from an epidemic in coastal karnataka. Indian J Dermatol. 2011;56(3):290–4. doi: 10.4103/0019-5154.82483 21772590 PMC3132906

[13] Inamadar AC, Palit A, Sampagavi VV, Raghunath S, Deshmukh NS. Cutaneous manifestations of chikungunya fever: observations made during a recent outbreak in south India. Int J Dermatol. 2008;47(2):154–9. doi: 10.1111/j.1365-4632.2008.03478.x 18211486

[14] Kannan M, Rajendran R, Sunish IP, Balasubramaniam R, Arunachalam N, Paramasivan R, et al. A study on chikungunya outbreak during 2007 in Kerala, South India Indian J Med Res. 2009;129(3):311–315. Available from: http://citeseerx.ist.psu.edu/viewdoc/download?doi=10.1.1.548.1861&rep=rep1&type=pdf. 19491425

[15] Katti R, Shahapur PR, Udapudi KL. Impact of chikungunya virus infection on oral health status: an observational study. Indian J Dent Res. 2011;22(4):613. doi: 10.4103/0970-9290.90325 22124070

[16] Kumar R, Sharma MK, Jain SK, Yadav SK, Singhal AK. Cutaneous manifestations of chikungunya fever: observations from an outbreak at a Tertiary Care Hospital in Southeast Rajasthan, India. Indian Dermatol Online J. 2017;8(5):336–42. doi: 10.4103/idoj.IDOJ_429_16 28979866 PMC5621193

[17] Paul BJ, Pannarkady G, Moni SP, Thachil EJ. Clinical profile and long-term sequelae of Chikungunya fever. Indian J Rheumatol. 2011;6(1):12–9. doi: 10.1016/S0973-3698(11)60024-1

[18] Riyaz N, Riyaz A, Rahima ALEN, Anitha PM, Aravindan KP, et al. Cutaneous manifestations of chikungunya during a recent epidemic in Calicut, north Kerala, south India. Indian J Dermatol Venereol Leprol. 2010;76(6):671–6. doi: 10.4103/0378-6323.72466 21079311

[19] Shruti M, D MK, Singh KP, Dhole TN. Emergence of Chikungunya infection in North India. Ann Pathol Lab Med. 2016;3(4):314–9. Available from: https://www.pacificejournals.com/journal/index.php/apalm/article/view/apalm782/pdf_229.

[20] Suryawanshi SD, Dube AH, Khadse RK, Jalgaonkar SV, Sathe PS, Zawar SD, et al. Clinical profile of chikungunya fever in patients in a tertiary care centre in Maharashtra, India. Indian J Med Res. 2009;129(4):438–41. . 19535840

[21] Vijayakumar KP, Nair Anish TS, George B, Lawrence T, Muthukkutty SC, Ramachandran R. Clinical Profile of Chikungunya Patients during the Epidemic of 2007 in Kerala, India. J Glob Infect Dis. 2011;3(3):221–6. doi: 10.4103/0974-777X.83526 21887052 PMC3162807

[22] Borgherini G, Poubeau P, Staikowsky F, Lory M, Le N, Becquart JP, et al. Outbreak of chikungunya on Reunion Island: early clinical and laboratory features in 157 adult patients. Clin Infect Dis. 2007;44(11):1401–7. doi: 10.1086/517537 17479933

[23] Robin S, Ramful D, Zettor J, Benhamou L, Jaffar-Bandjee MC, Rivière JP, et al. Severe bullous skin lesions associated with Chikungunya virus infection in small infants. Eur J Pediatr. 2010;169(1):67–72. doi: 10.1007/s00431-009-0986-0 19401826

[24] Staikowsky F, Talarmin F, Grivard P, Souab A, Schuffenecker I, Le Roux K, et al. Prospective study of Chikungunya virus acute infection in the Island of La Réunion during the 2005–2006 outbreak. PLoS ONE. 2009;4(10):e7603. doi: 10.1371/journal.pone.0007603 19893613 PMC2764049

[25] Talarmin F, Staïkowsky F, Schoenlaub P, Risbourg A, Nicolas X, Zagnoli A, et al. Manifestations cutanéo-muqueuses de l’infection par le virus chikungunya chez l’adulte à la Réunion [Skin and mucosal manifestations of chikungunya virus infection in adults in Reunion Island]. Med Trop (Mars). 2007;67(2):167–73. French. . 17691437

[26] Razmy AM. Clinical features of chikungunya infection in Sri Lanka. Asian Pac J Trop Dis. 2014;4(2):131–4. doi: 10.1016/S2222-1808(14)60329-7

[27] Taubitz W, Cramer JP, Kapaun A, Pfeffer M, Drosten C, Dobler G, et al. T. Chikungunya fever in travelers: clinical presentation and course. Clin Infect Dis. 2007;45(1):e1–4. doi: 10.1086/518701 17554689

[28] Simon F, Parola P, Grandadam M, Fourcade S, Oliver M, Brouqui P, et al. Chikungunya infection: an emerging rheumatism among travelers returned from Indian Ocean islands. Report of 47 cases. Medicine (Baltimore). 2007;86(3):123–37. doi: 10.1097/MD/0b013e31806010a5 17505252

[29] Chang AY, Encinales L, Porras A, Pacheco N, Reid SP, Martins KAO, et al. Frequency of Chronic Joint Pain Following Chikungunya Virus Infection: A Colombian Cohort Study. Arthritis Rheumatol. 2018;70(4):578–84. doi: 10.1002/art.40384 29266783 PMC7928267

[30] Heath CJ, Lowther J, Noël TP, Mark-George I, Boothroyd DB, Mitchell G, et al. The identification of risk factors for chronic chikungunya in Grenada, West Indies: A Cross-Sectional Cohort Study. Open Forum Infect Dis. 2018;5(1):ofx234. doi: 10.1093/ofid/ofx234 29308412 PMC5753193

[31] Deeba IM, Hasan MM, Al A, Siam MHB, Islam MS, Raheem E, et al. Manifestations of Atypical Symptoms of Chikungunya during the Dhaka Outbreak (2017) in Bangladesh. Am J Trop Med Hyg. 2019;100(6):1545–8. doi: 10.4269/ajtmh.19-0122 31038100 PMC6553908

[32] Casais PM, Akrami K, Cerqueira-Silva T, Moraes LP, Rigaud VN, Neto ES, et al. Oral lesions are frequent in patients with Chikungunya infection. J Travel Med. 2020;27(4):taaa040. doi: 10.1093/jtm/taaa040 32186714 PMC7359922

[33] Doria YCS. Profile of stomatognathic manifestations in adults in the chronic phase of chikungunhya. Master Thesis. CESMAC University Center; 2019.

[34] Fatima K, Bana MA, Khalid A, Kadri WB. Oral manifestations of chickengunya and gender difference among the patients of industrial area of Karachi, Pakistan. Rawal Med J. 2020;45(3):527–30.

[35] Gardner J, Rudd PA, Prow NA, Belarbi E, Roques P, Larcher T, et al. Infectious chikungunya virus in the saliva of mice, monkeys and humans. PLoS ONE. 2015;10(10):e0139481. doi: 10.1371/journal.pone.0139481 26447467 PMC4598147

[36] Pedrosa MS, de Paiva MHP, LGFL O, Pereira SMS, da Silva CHV, Pompeu JGFP. Oral manifestations related to dengue fever: a systematic review of the literature. Aust Dent J. 2017;62:404–411. doi: 10.1111/adj.12516 28379606

[37] Lokireddy S, Vemula S, Vadde R. Connective tissue metabolism in chikungunya patients. Virol J. 2008;5:31. doi: 10.1186/1743-422X-5-31 18302795 PMC2291039

