# Supplementary material for: Oral manifestations in chikungunya patients: A systematic review
Source: PLoS Negl Trop Dis. 2021 Jun 10;15(6):e0009401. doi: 10.1371/journal.pntd.0009401 (PMC8191910; doi:10.1371/journal.pntd.0009401)
Supplement: S1 Table — (DOCX) [file pntd.0009401.s001.docx]

| **S1_Table. Search strategies** | | | |
| --- | --- | --- | --- |
| **Search Date** | **Data base** | **Strategy used** | **Number of Studies** |
| January 16, 2021 | MEDLINE (PubMed) | ("Chikungunya Fever"[Mesh] OR "Chikungunya Virus Infection" OR "Chickungunya Fever" OR "Chikungunya virus"[Mesh] OR "Chikungunya" OR "Chickungunya" OR "CHIK" OR "CHIKV") AND ("Oral Health"[Mesh] OR "Oral Health" OR "Mouth"[Mesh] OR "Mouth" OR "Oral Cavity" OR "Cavitas Oris" OR "Vestibule of the Mouth" OR "Oral Cavity Proper" OR "Cavitas oris propria" OR "Oral Manifestations"[Mesh] OR "Oral Manifestation" OR "Mouth Diseases"[Mesh] OR "Mouth Disease" OR "Mouth Mucosa"[Mesh] OR "Mouth Mucosa" OR "Oral Mucosa" OR "Mucosa, Oral" OR "Buccal Mucosa" OR "Oral Hemorrhage"[Mesh] OR "Oral Ulcer"[Mesh] OR "Oral Ulcer" OR "Mouth Ulcer" OR "Oral lesion" OR "Oral lesions" OR "Stomatitis, Aphthous"[Mesh] OR "Aphthous Stomatitides" OR "Aphthous Stomatitis" OR "Aphthous Ulcer" OR "Aphthae" OR "Canker Sore" OR "Periadenitis Mucosa Necrotica Recurrens" OR "Burning Mouth Syndrome"[Mesh] OR "Deglutition Disorders"[Mesh] OR "Deglutition Disorder" OR "Swallowing Disorder" OR "Dysphagia" OR "Oropharyngeal Dysphagia" OR "Esophageal Dysphagia" OR "Stomatitis"[Mesh] OR Stomatitides OR "Oral Mucositis" OR "Oromucositis" OR "Xerostomia"[Mesh] OR "Hyposalivation" OR "Hyposalivations" OR "Asialia" OR "Mouth Dryness" OR "Sialorrhea"[Mesh] OR "Hypersalivation" OR "Drooling" OR "Erythema"[Mesh] OR "Dysgeusia"[Mesh] OR "Distorted Taste" OR "Altered Taste" OR "Parageusia" OR "Distaste" OR "Trismus"[Mesh] OR "Lockjaw" OR "Lock Jaw" OR "Masseter Muscle Spasm" OR "Masseter Spasm" OR "Temporomandibular Joint Disorders"[Mesh] OR "Temporomandibular Joint Disorder" OR "TMJ Disorder" OR "Temporomandibular Disorder" OR "Temporomandibular Joint Disease" OR "TMJ Disease" OR "Chronic joint pain" OR "Chronic polyarthritis" OR "Smaller joints" OR "Small joints" OR "Palate"[Mesh] OR "Palates" OR "Incisive Papilla" OR "Palate, Hard"[Mesh] OR "Hard Palate" OR "Palatine Bone" OR "Palate, Soft"[Mesh] OR "Velum Palatinum" OR "Soft Palate" OR "Tongue"[Mesh] OR "Tongue, Fissured"[Mesh] OR "Fissured Tongue" OR "Lingua Plicata" OR "Furrowed Tongue" OR "Lip"[Mesh] OR "Philtrum" OR "Gingiva"[Mesh] OR "Gum" OR "Interdental Papilla" OR "Mouth Floor"[Mesh] OR "Sublingual Region" OR "Floor of Mouth" OR "Cheek"[Mesh] OR "Cheeks" OR "Bucca" OR "Dental Plaque"[Mesh] OR "Glossitis, Benign Migratory"[Mesh] OR "Benign Migratory Glossitis" OR "Geographic Tongue" OR "Mucocutaneous" OR "Cutaneous" OR "Bitter taste" OR Glossitis OR "Gingival bleeding" OR "Hemorrhagic signs" OR "Gingival edema" OR "Gingival pain" OR "Oral mucosal burning" OR "Burning sensation" OR "Oral mucosal erythema" OR "Gingival erythema" OR "Bleeding gum" OR "Gum pain" OR "gingivitis" OR "Oral pain" OR "Excess salivation" OR "Tooth loss" OR "Atypical symptoms" OR "Differential diagnosis") | 412 |
| January 16, 2021 | The Cochrane Library | # 1- MeSH descriptor: [Chikungunya Fever] explodes all trees  # 2- MeSH descriptor: [Chikungunya virus] explodes all trees  #3- #1 AND #2 | 25 |
| January 16, 2021 | SCOPUS | ( ( TITLE-ABS-KEY ( "Chikungunya Fever" ) OR TITLE-ABS-KEY ( "Chikungunya virus" ) ) ) AND ( ( TITLE-ABS-KEY ( "Oral Manifestation" ) OR TITLE-ABS-KEY ( deglutition AND disorders ) OR TITLE-ABS-KEY ( "Oral Ulcer" ) OR TITLE-ABS-KEY ( dysgeusia ) OR TITLE-ABS-KEY ( stomatitis ) ) ) | 62 |
| January 16, 2021 | BVS | ((Chikungunya Fever) OR (Chikungunya virus)) AND (Oral) | 86 |
| January 16, 2021 | EMBASE | ('chikungunya'/exp OR 'chikungunya virus'/exp) AND ('oral mucosal disease'/exp OR 'mouth disease'/exp OR 'stomatognathic system'/exp) | 206 |
| January 16, 2021 | CAPES | chikungunya and oral health | 04 |
