# Supplementary material for: Oral manifestations in chikungunya patients: A systematic review
Source: PLoS Negl Trop Dis. 2021 Jun 10;15(6):e0009401. doi: 10.1371/journal.pntd.0009401 (PMC8191910; doi:10.1371/journal.pntd.0009401)
Supplement: S2 Table — (DOCX) [file pntd.0009401.s002.docx]

**S2_Table. Data extraction**

| **1st author (year) [ref]** | **Country and year of study** | **Study Design** | **N Total / Gender** | **Age** | **Diagnosis type/ Date extraction type** | **Oral manifestations** | **Comments** |
| --- | --- | --- | --- | --- | --- | --- | --- |
| Anshul et al. (2020) | Índia  (Aug-Nov2016) | Prospective observational cohort study | N=150  59 males and 91 females | 1 month to 85 years | 150 suspected chikungunya  Only clinical examination | - 9 cases (6%) of aphthous ulcer - 9 cases (6%) of angular cheilitis | Mucosal lesions lasted for 7-10 days and subsided completely without any sequelae. |
| Bandyopadhyay  et al. (2008 ) | India (2007) | Observational historical cohort study | N=26  13 males and 13 females | 10 to 60 years | 26 confirmed chikungunya  Clinical, laboratorial examinations and self-reported (anamnesis) | - 1 case (3.8%) of oral ulcers - 1 case (3.8%) lips herpes | Contrary to other studies that showed high prevalence of oral mucosa aphthous ulcers, in the present study the author pointed out that this type of outcome was observed in only 1 case. |
| Bhat et al. (2011) | India  (Jun-Aug 2008) | Observational historical cohort study | N=75  37 males and 38 females | 1 to 70 years | 75 suspected chikungunya  Clinical, laboratorial examinations and self-reported (anamnesis) | - 12 cases (16%) of oral ulcers | Oral ulcers were observed in both sexes, predominantly in the acute phase of the disease (2 to 7 days), presenting as multiple small ulcers (2 to 5 mm) with inflammatory aspect, painful and covered by plaque. |
| Borgherini  et al. (2007) | Reunion island  (Mar 2005 - Apr 2006) | Observaciona historical cohort study | N=157  87 males and 70 females | 16 years and over | 157 confirmed chikungunya (only acute phase)  Medical records and laboratorial examination | - 4 cases (2.5%) of oral ulcers - 2 cases (1.25%) of gingival bleeding | On Reunion Island, although the life expectancy at birth (75.5 years) the age distribution is quite different with the majority of the population (65%) aged <40 years. Considering these data and the mean age of the included patients, we could conclude that there is a major selection bias, because most of the observed cases occurred in persons aged >40 years.  In 97 cases, hospitalizations occurred due to clinical complications resulting from the association of chikungunya with comorbidities such as hypertension, diabetes, ischemic heart disease, pulmonary and renal insufficiency. Five deaths were observed. |
| Casais et al. (2020) | Brasil  (March 2016 – Jun 2017) | Prospective observational cohort study | N=105  41 male and 64 female | 28 to 57 years | 105 confirmed chikungunya  Clinical and laboratory examinations | - 20 cases (19%) of dysfagia - 15 cases (14%) of oral ulcers - 7 cases (6,6%) of gingival bleeding - 3 cases (2,8%) of herpes - 1 case (1%) of gingival edema | Was detected CHIKV in saliva of 27% of patients with oral involvement suggesting that lesions may result from direct viral activity. The presence of oral ulcers was found to be relatively common during acute CHIKV infection, resulting in pain and dysphagia that potentially increases the morbidity of this arbovirus infection. |
| Chang et al. (2018) | Colombia  (20-month segment from Jan 2015) | Prospective observational cohort study | N=485  97 males and 388 females | with a mean age of 49.1 years. | 485 confirmed chikungunya  (only chronic phase)  Medical records, laboratorial examination and self-reports (telephone survey) | - 6 cases (1.23%) of gingival bleeding | Comparative study between chronic cases with persistent or intermittent joint pain. There was no statistical difference (p=0,65) between joint pain and gingival bleeding  Significant (P < 0.002)  The most common baseline comorbidities were 57 cases (12%) of hypertension and 34 cases (7%) of diabetes; 17 patients (4%) reported previous history of arthritis. |
| Deeba et al. (2019) | Bangladesh  (2017) | Cross-sectional observational study | N= 1089  612 males and 486 females | aged <15 to> 60 | 1089 cases. Not reported the diagnosis type  (only acute phase)  Medical records and self-reported (questionnaire and interview) | - 414 cases (37.7%) of oral ulcers | This study reported the frequency of atypical symptoms in the Dhaka (2017) outbreak. Over 70% of patients reported more than one atypical symptom during the acute phase of the disease.  A total of 336 (30.6%) patients had comorbidities, the most common being hypertension (12.6%), diabetes (9.4%) and ischemic heart disease (3%). |
| Doria (2019) | Brazil  (2015-2016) | Cross-section observational study | N=40  4 males and 36 females | 20 to 68 years | 40 confirmed chikungunya  (only chronic phase)  Clinical, laboratorial examinations and self-reported (questionnaire) | - 4 cases (10%) of TMJ arthralgia - 3 cases (7.5%) of bitter taste in the mouth - 10 cases (25%) of oral ulcers - 8 cases (20%) of cervical lymphadenopathy - candidiasis (number of cases not reported) | According to the prevalence of age and gender for the chronic phase of the disease. the prevalence of recurrent thrush and TMJ dysfunction was predominantly found in females and those over 45 years of age |
| Fatima et al. (2020) | Pakistan (Jul –Dec 2018) | Cross-sectional observational study | N=531  200 M  331 F | 2 to >75 years | 531 confirmed chikungunya  Medical records, clinical examination and self-reported (anamnesis) | -56 cases (10.5%) of oral ulcers  -63 cases (11.8%) of TMJ arthralgia  - 261 cases (49.1%) of mouth opening pain  -100 cases (18.8%) of gingival edema  -51 cases (9.6%) of cervical lymphadenopathy | Females were affected more as compare to male gender with oral manifestation and oral symptoms were observed more in patient of 50 years and above |
| Gardner  et al. (2015) | Nicaragua  (Aug 2014 - Jun 2015) | Prospective observational cohort study | N=13  9 males and 4 females | 7 to 14 years | 13 confirmed chikungunya  (only acute phase)  Medical records, clinical examination, and self-reported (anamnesis) | - 3 cases (21.4%) of gingival bleeding | This study pointed out the possibility of the presence of active chikungunya virus in the saliva of human patients in the acute phase of the disease, providing support for the claim that the viral agent in saliva may be associated with hemorrhagic lesions in the nasal / oral cavities during the viremic period; and which of the safety implications of infectious saliva may be relevant to dentistry |
| Heath et al. (2018) | Grenada  (Nov 2015 - Jan 2016) | Cross-sectional ~~cohor~~t observacional study | N=240  64 males and 176 females | 4 to 89 years | 240 confirmed chikungunya  (only chronic phase)  Clinical, laboratorial examinations and self-reported (interview) | - 87 cases (36.25%) of bitter taste in the mouth - 9 cases (3.7%) of gingival bleeding | This study describes the widespread morbidity that the CHIKV epidemic had on the population of Grenada, and our data demonstrate that chronic CHIKV disease affects people across the ethnic and socioeconomic spectrum. Increasing age was found to be a significant risk factor for chronic CHIKV arthralgia in our cohort, with those in the 25- to 44-year-old age group at the highest risk.  Comorbidities associated: Prior Dengue Infection, Asthma, Respiratory and Cardiovascular Diseases, Hypertension, Diabetes, High Cholesterol, Seizure Disorders and Cancer |
| Inamadar et al. (2008) | India  (May - Jun 2006) | Prospective observational cohort study | N=145  93 males and 52 females | 25 days to 85 years | 145 suspected chikungunya  Only clinical examination | - 1 case (0.68%) of tongue and palate hyperpigmentation - 1 case (0.68%) of oral ulcers - 1 case (0.68%) lip depigmentation | According to the authors, most of the skin changes observed in this study had not previously been reported. These findings could be attributed to the new (African) genotype of the virus that caused this outbreak in India, in contrast with previous outbreaks caused by the Asian genotype |
| Kannan et al. (2009) | India  (2007) | Cross-sectional ~~cohort~~ observacional study | N=354  160 males and 194 females | 1 to 45 years | 354 suspected chikungunya  Medical records and self-reported (anamnesis) | - 63 cases (17.8%) of oral ulcers - 5 cases (1.4%) of gingival bleeding | No gender difference was observed for any of the symptoms. Regarding age ranges and outcomes, a higher prevalence was observed with increasing age.  1-15 years - N = 49  4 cases (8.2%) of oral ulcers;  16-35 years - N = 111  17 cases (15.3%) of oral ulcers and 2 cases (1.8%) of gingival bleeding;  36-45 years - N = 90  19 cases of oral ulcers and 1 case of gingival bleeding;  > 45 years - N = 104  23 cases of oral ulcers and 2 cases of gingival bleeding |
| Katti et al. (2011) | India  (Jun - Sep 2006) | Prospective observational cohort study | N=97  47 males and 50 females | 17 to 51 years | 97 confirmed chikungunya  Clinical, laboratorial examinations and self-reported (anamnesis) | - 56 cases (54.32%) of gingival pain - 56 cases (54.32%) of gingival bleeding - 56 cases (54, 32%) of gingival burning - 32 cases (29.1%) of chewing difficulty - 32 cases (29.1%) of dysphagia - 22 cases (21.34%) of halitosis - 18 cases (17.46%) of oral ulcers - 12 cases (11.64%) of mouth opening pain - 6 cases (6%) of TMJ arthralgia - 10 cases (9.7%) of excess salivation - 8 cases (7.76%) of whitish plaque deposit in the gums - 1 case (0.97%) of tooth loss | Gingival pain was observed almost equally among younger individuals with 25 cases (64.1%) and older age 19 cases (67.85%). Gingival burning was more common among the younger age group with 28 cases (71.79%). Symptoms such as gingival bleeding with 26 cases (96.42%), inability to chew with 11 cases (39.28%) and halitosis with 8 cases (28.57%) were more pronounced in the age group over 45 years.   Statistically significant association was found in the gingival plaque index scores (χ2 = 6.417, P = 0.040). Severe gingivitis was observed in patients with chronic disease, while among patients with acute disease, moderate gingivitis was observed. No significant association was observed regarding oral hygiene status (χ2 = 2.166, P = 0.347) and plaque scores (χ2 = 4.125, P = 0.127).   Gingival pain was most commonly observed among patients with chronic disease, while chewing pain was frequently seen in patients with acute disease. However, no statistically significant association was observed. |
| Kumar et al*.*(2017) | India  (Jul - Oct 2016) | Prospective observational cohort study | N=112  62 males and 50 females | 1 month to 77 years. | 76 confirmed chikungunya  Clinical and laboratorial examinations | - 4 cases (3.6%) of angular cheilitis - 11 cases (9.8%) of oral ulcers - 2 cases (1.8%) of hard palate hyperpigmentation | Oral ulcers subsided within 3–5 days after the appearance. |
| Paul et al. (2011) | India  (Jun - Dec 2007) | Prospective observational cohort study | N=100  40 males and 60 females | 20 to 60 years | 100 confirmed chikungunya  Clinical, laboratorial examinations and self-reported (telephone interview) | - 45 cases (45%) of oral ulcers - 17 cases (17%) of glossitis | The ulcers were very painful and severe in some of them and appeared during the acute phase of the disease. |
| Razmy et al. (2014) | Sri Lanka  (2006) | Prospective observational cohort study | N=789  318 males and 470 females | < 1 to > 63 | 789 suspected chikungunya  Only self-reports (questionnaire) | - 12 cases (1.5%) of gingival bleeding - 73 cases (9.3%) of oral ulcers (9.3%) | 1.5% of patients had gingival bleeding with gender association (χ2 = 5.688, P = 0.02). About 2.6% of female patients had gingival bleeding and only 0.5% in males.  9.3% of patients had a gender-associated mouth ulcer (χ2 = 17.664, P <0.000). About 13.7% of female patients had mouth ulcers and this occurred in only 5.0% for males. No significant association was found between age and gingival bleeding (χ2 = 2.435, P = 0.51) |
| Riyaz et al. (2010) | India  (Jul - Sep 2009) | Prospective observational cohort study | N=157  63 males and 99 females | 1 month to 78 years | 157 confirmed chikungunya  Clinical and laboratorial examinations | - 22 cases (13.64%) of oral ulcers - cheilitis (Not reported the number of cases) | Oral manifestations lasted 7 to 10 days and disappeared without sequelae. |
| Robin et al. (2010) | Reunion island  (Mar 2005 - Oct 2006) | Observational historical cohort study | N=13  8 males and 5 females | under 6 months | 13 confirmed chikungunya  Medical records, clinical, laboratorial examinations and self-reported (anamnesis) | - 3 cases (23%) of oral ulcers - 1 case (7.6%) of cervical lymphadenopathy | This study described pediatric cases of laboratory confirmed CHIKV infection associated with severe and extensive bullous skin lesions. None of these infants had history of cutaneous disease |
| Shruti et al*.*(2016) | India  (Sep 2010 - Aug 2011) | Prospective observational cohort study | N=248  Group 1  N=204  131 males and 73 females  Group 2  N=44  13 males and 31 females | all age groups | Group 1  204 not confirmed chikungunya  Group 2  44 confirmed chikungunya  Clinical, laboratorial examinations and self-reported (anamnesis) | - Group 1 - 48 cases (23.5%) of oral ulcers - Group 2 - 16 cases (36.5%) of oral ulcers | The main clinical features in the present study were fever with joint pain, rash and aphthous ulcers on oral mucosa and tongue) |
| Simon et al. (2007) | France  (Feb 2005 - Apr 2006) | Prospective observational cohort study | N=44  25 males and 22 females | 6 months to 73 years | 44 confirmed chikungunya  Clinical, laboratorial and self-reported examinations (telephone interview and anamnesis) | - 2 cases (4.2%) of gingival bleeding - 7 cases (14.9%) of TMJ arthralgia - 1 case (2.1%) of lip herpes - 1 case (2.1%) of oral ulcers - 1 case (2.1%) of dysgeusia | It was a study on the clinical aspects of CHIKV infection imported to Marseilles, France, in travelers returning from the Indian Ocean Islands.  Patients with numerous comorbidities such as hypertension, diabetes, heart failure, hyperthyroidism, obesity, psoriasis, and pre-existing joint problems |
| Singaraju et al*.*(2010) | India  (Jan - Mar 2010) | Prospective observational cohort study | N=110  Group 1  N=110  47 males and 63 females  Group 2  N=37  18 males and 19 females | 1 to 50 years | Group 1  110 suspected chikungunya  Group 2  37 confirmed chikungunya  Clinical, laboratorial examinations and self-reported (questionnaire and anamnesis) | - Group 1 - 106 cases (96.37%) of oral manifestations - 106 cases (96.37%) of oral mucosal burning - 103cases (93.63%) of oral mucosal erythema - 79 cases (71.81%) of oral ulcers - 53 cases (48.18%) of dysphagia - 63 cases (57.27%) of TMJ arthralgia - 85 cases (77.27%) of gingivitis - 85 cases (77.27%) of cervical lymphadenopathy - 8 cases (7.27%) of interdental mucosa desquamation - Group 2 - 37 cases (100%) of oral manifestations - 37 cases (100%) of oral mucosal burning - 34 cases (91.89%) of oral mucosa erythema - 32 cases (86.48%) of oral ulcers - 26 cases (70.27%) of dysphagia - 31 cases (83.78%) of TMJ arthralgia - 28 cases (75.67%) of gingivitis - 30 cases (81.08%) of cervical lymphadenopathy - 7 cases (18.91%) of interdental mucosa scaling | Comparisons were made between suspected and confirmed patients and oral manifestations in relation to the following age ranges:    Group 1  1-20 years (N = 16)  Higher prevalence of trismus (81.25%) and cervical lymphadenopathy (93.25%)  36-50 years (N = 30)  Higher prevalence of ulcerations (76.66%) and gingivitis (83.33%)  > 50 years (N = 42)  Higher prevalence of oral mucosal burning (97.62%), erythema (95.23%), dysphagia (83.33%) and distaste (85.75 %)    Group 2  1-20 years (N = 4)  Higher prevalence of erythema (100%), trismus (100%) and cervical lymphadenopathy (100%)  36-50 years (N = 10)  Higher prevalence of ulcerations (90%) and gingivitis (80%)  > 50 years (N = 16)  Higher prevalence of dysphagia (75%) and dysphagia (75%)   Oral manifestations generally occurred 1-2 days before or after the onset of the acute phase of the disease.  Erythema-like manifestations were found on the lips, tongue, floor of the mouth and palate. Multiple ulcers were found in the soft palate, hard palate, tongue and floor of the mouth   The authors proposed the introduction of the term "Gunya stomatitis", representing the clinical condition of TMJ arthralgia, associated with generalized erythema of the mucosa and ulcers in the oral cavity with concomitant dysphagia. |
| Staikowsky et al. (2009) | Reunion island  (Mar - May 2006) | Prospective observational cohort study | N=260  131 males and 129 females  Group A1  N=180  96 males and 84 females  Group A2  N=34  13 males and 21 females  Group B  N=46  22 males and 24 females | 15 to 96 years.    Group A1:  15 to 96 years    Group A2:  17 to 89 years    Group B:  16 to 93 years | 214 confirmed chikungunya  Group A1  180 cases in the acute phase  Group 2  34 cases in the chronic phase  Group B  46 not confirmed chikungunya  Clinical, laboratorial examinations and self-reported (questionnaire) | - Suspected Cases N=260 - 76 cases (29.4%) of dysgeusia - 6 cases (2.07%) of gingival bleeding - 5 cases (1.86%) of TMJ arthralgia - 1 case (0.46%) of oral ulcers   Group A1   - 46 cases (40.4%) of dysgeusia - 2 cases (1.1%) of gingival bleeding - 3 cases (1.8%) of TMJ arthralgia - Group A2 - 17 cases (58.6%) of dysgeusia - 3 cases (8.8%) of gingival bleeding - 1 case (3.2%) of TMJ arthralgia - 1 case (2.9% 0) of oral ulcer - Group B - 13 cases (35.1%) of dysgeusia - 1 case (2.2%) of gingival bleeding - 1 case (2.4%) of TMJ arthralgia | The study compared the signs and symptoms of the disease chikungunya among the 3 groups.  Associated comorbidities presented:  hypertension, diabetes, obesity, heart disease, kidney disease, lung disease, stroke, and epilepsy.  Factors influencing disease severity are dominated by age and comorbidities.  The study recorded rare locations of arthralgia, such as of the temporomandibular joint |
| Suryawanshi et al*.*(2009) | India  (Jul - Sep 2006) | Prospective observational cohort study | N= 405  Group 1  N=318  Not reporter gender  Group 2  N=87  61 males and 26 females | 13-70 years | Group 1  318 suspected chikungunya  Group 2  87 confirmed chikungunya  Clinical, laboratorial examinations and self-reported (anamnesis) | Group 1   - 31 cases (10%) of oral ulcers - 31 cases (10%) of gingivitis 4 cases (1.25%) of cervical lymphadenopathy   Group 2   - 9 cases (10.34%) of oral ulcers - 9 cases (10.34%) of gingivitis - 8 cases (9.19%) of cervical lymphadenopathy | They considered cervical lymphadenopathy and oral ulcers as rare manifestations reported in the study |
| Talarmin et al. (2007) | Reunion island  (Mar - Apr 2006) | Prospective observational cohort study | N=212  105 males and 107 females | 15 to94 years | 212 confirmed chikungunya  Clinical, laboratorial examinations and self-reported (anamnesis) | - 2 cases (0.94%) of gingival bleeding - 62 cases (29.2%) of dysgeusia - Glossitis with tongue decapilation (not reported the number of cases) - Oral mucosa erythema (not reported the number of cases) - Bitter taste (not reported the number of cases) | Mucosal involvement is frequent: pharyngitis, mouth ulcers, glossitis with decapilation of the tongue.  Dysgeusia, a bitter or metallic taste, was a good sign for diagnostic guidance |
| Taubitz et al*.*(2007) | Germany  (Jan - Oct 2006) | Observational study of historical cohort | N=20  6 males and 14 females | 12 to 64 years. | 20 confirmed chikungunya  Clinical, laboratorial examinations and self-reported (anamnesis)) | - 1 case (5%) of gingival bleeding | This study was conducted to investigate travelers who are returning from countries of epidemicity and who present with such symptoms  and reported a history compatible with CHIKV infection. |
| Vijayakumar et al*. (*2011) | India  (Oct - Nov 2007) | Cross-section observational study | N=1913  945 males and 968 females | 1 to > 60 years | 1913 suspected chikungunya  Self-reported (interview and questionnaire) | - 382 cases (20%) of oral ulcers | Oral ulcers occurred mainly in the first week of the disease. |
