# Supplementary material for: Oral manifestations in chikungunya patients: A systematic review
Source: PLoS Negl Trop Dis. 2021 Jun 10;15(6):e0009401. doi: 10.1371/journal.pntd.0009401 (PMC8191910; doi:10.1371/journal.pntd.0009401)
Supplement: S3 Table — (DOCX) [file pntd.0009401.s003.docx]

**S3_Table. Newcastle-Ottawa risk of bias tool for cohort studies**

|  | Representativeness of the exposed cohort | Selection of the non-exposed cohort | Ascertainment of exposure | Demonstration that outcome of interest was not present at start of study | Comparability of cohorts on the basis of the design or analysis | Assessment of outcome | Was follow-up long enough for outcomes to occur | Adequacy of follow up of cohorts | Total |
| --- | --- | --- | --- | --- | --- | --- | --- | --- | --- |
| Anshul et al. (2020) |  |  | * |  | * | * | * | * | 5 |
| Bandyopadhyay et al. (2008) |  |  | * |  | * | * | * | * | 5 |
| Bhat et al. (2011) |  |  | * |  | * | * | * | * | 5 |
| Borgherini  et al. (2007) |  |  | * |  | * | * | * | * | 5 |
| Casais et al (2020) |  |  | * |  | * | * | * | * | 5 |
| Chang et al. (2018) |  |  | * |  | * | * | * | * | 5 |
| Gardner  et al. (2015) |  |  | * |  | * | * | * | * | 5 |
| Inamadar et al*.* (2008) |  |  | * |  | * | * | * | * | 6 |
| Katti *et al.* (2011) |  |  | * |  | * | * | * | * | 5 |
| Kumar et al. (2017) |  |  | * |  | * | * | * | * | 5 |
| Paul et al. (2011) |  |  | * |  | * | * | * | * | 5 |
| Razmy et al. ( 2014) | * |  | * |  | * | * | * | * | 6 |
| Riyaz et al. (2010) |  |  | * |  | * | * | * | * | 5 |
| Robin et al. (2010) |  |  | * |  | * | * | * | * | 5 |
| Shruti et al*.* ( 2016) |  | * | * |  | ** | * | * | * | 7 |
| Simon et al. (2007) |  |  | * |  |  | * | * | * | 5 |
| Singaraju et al*.*(2010) |  | * | * |  | * | * | * | * | 7 |
| Staikowsky et al. (2009) |  | * | * |  | * | * | * | * | 7 |
| Suryawanshi et al. (2009) |  | * | * |  | * | * | * | * | 7 |
| Talarmin et al*.*(2007) |  |  | * |  | * | * | * | * | 5 |
| Taubitz et al. (2007) |  |  | * |  | * | * | * | * | 5 |

**Scale cohort studies: Selection:**1) Representativeness of the exposed cohort^⁕^ a) truly representative of the average in the community, b) somewhat representative of the average community; 2) Selection of the non-exposed cohort a) drawn from the same community as the exposed cohort^⁕;^ 3) Ascertainment of exposure^⁕^ a) secure records), b) structured interview; 4) Demonstration that outcome of interest was not present at start of study a) yes^⁕^; **Comparability:** 1) Comparability of cohorts on the basis of the design or analysis a) study controls for most important factor⁕, b) study controls for any additional factor^⁕^; **Outcome:** 1) Assessment of outcome^⁕^ a) independent blind assessment, b) record linkage; 2) Was follow-up long enough for outcomes to occur a) yes^⁕^, 3) Adequacy of follow up of cohorts^⁕^ a) complete follow up - all subjects accounted for; b) subjects lost to follow up unlikely to introduce bias - small number lost . A study can be awarded a maximum of one star for each numbered item within the Selection and Exposure categories. A maximum of two stars can be given for Comparability.
