# Supplementary material for: Oral manifestations in chikungunya patients: A systematic review
Source: PLoS Negl Trop Dis. 2021 Jun 10;15(6):e0009401. doi: 10.1371/journal.pntd.0009401 (PMC8191910; doi:10.1371/journal.pntd.0009401)
Supplement: S4 Table — (DOCX) [file pntd.0009401.s004.docx]

**S4_Table. Newcastle-Ottawa risk of bias tool for cross-sectional studies**

|  | Representativeness of the sample | Sample size | Non-respondents | Ascertainment of the exposure  (risk factor) | Comparability | Assessment of outcome | Statistical test | Total |
| --- | --- | --- | --- | --- | --- | --- | --- | --- |
| Deeba et al*.* (2019) | * | * |  | * | * | * | * | 6 |
| Doria (2019) |  | * |  | ** | * | * | * | 6 |
| Fatima et al. (2020) | * | * |  | * | * | * | * | 6 |
| Heath et al. (2018) |  | * |  | ** | ** | * | * | 6 |
| Kannan et al. (2009) | * | * |  | * | * | * | * | 6 |
| Vijayakumar et al. (2011) | * | * |  | * | * | * | * | 6 |

**Newcastle - Ottawa Quality Assessment Scale** (adapted for cross sectional studies)

Selection: (Maximum 5 stars) 1) Representativeness of the sample: a) Truly representative of the average in the target population. * (all subjects or random sampling) b) Somewhat representative of the average in the target population. * (nonrandom sampling) c) Selected group of users. d) No description of the sampling strategy. 2) Sample size: a) Justified and satisfactory. * b) Not justified. 3) Non-respondents: a) Comparability between respondents and non-respondents characteristics is established, and the response rate is satisfactory. * b) The response rate is unsatisfactory, or the comparability between respondents and non-respondents is unsatisfactory. c) No description of the response rate or the characteristics of the responders and the non-responders. 4) Ascertainment of the exposure (risk factor): a) Validated measurement tool. ** b) Non-validated measurement tool, but the tool is available or described.* c) No description of the measurement tool. Comparability: (Maximum 2 stars) 1) The subjects in different outcome groups are comparable, based on the study design or analysis. Confounding factors are controlled. a) The study controls for the most important factor (select one). * b) The study control for any additional factor. * Outcome: (Maximum 3 stars) 1) Assessment of the outcome: a) Independent blind assessment. ** b) Record linkage. ** c) Self report. * d) No description. 2) Statistical test: a) The statistical test used to analyze the data is clearly described and appropriate, and the measurement of the association is presented, including confidence intervals and the probability level (p value). * b) The statistical test is not appropriate, not described or incomplete.
