# Supplementary material for: Oral manifestations in chikungunya patients: A systematic review
Source: PLoS Negl Trop Dis. 2021 Jun 10;15(6):e0009401. doi: 10.1371/journal.pntd.0009401 (PMC8191910; doi:10.1371/journal.pntd.0009401)
Supplement: S5 Table — (DOCX) [file pntd.0009401.s005.docx]

**S5_Table. Excluded studies with reasons for exclusion**

| **No.** | **Complete Reference** | **Reason for exclusion** |
| --- | --- | --- |
| 1 | Agarwal J, Srivastava S, Shruti M, Singh KP, Singh M. A study on chikungunya virus infection in north India. Clin. Microbiol. Infect 2011; 17: S253. https://www.embase.com/search/results?subaction=viewrecord&id=L70599662&from=export U2 - L70599662 | The article contains partial reports of included study Shruti M, D MK, Singh KP, Dhole TN. Original Article Emergence of Chikungunya infection in North India. Ann Pathol Lab Med. 2016; 3 (4): 314–9. |
| 2 | Agarwal A, Vibha D, Srivastava A, Shukla G, Prasad K. Guillain-Barre syndrome complicating chikungunya virus infection. Journal of NeuroVirology. 2017; 23 (3); 504-7.  doi: 10.1007/s13365-017-0516-1. Epub 2017 Feb 13. PMID: 28194661. | The article is a case report. Design different from that established in the inclusion criterion: observational studies (case-control, cohort studies, cross-sectional studies). |
| 3 | Amorim, CS, Silva TR, Silva IM. Findings from the swallowing videofluoroscopy in a patient with chikungunya diagnosis. Dysphagia. 2018; 33(4): 535. https://doi.org/10.1007/s00455-018-9900-0 | The article is a case report. Design different from that established in the inclusion criterion: observational studies (case-control, cohort studies, cross-sectional studies). |
| 4 | Anderson KB, Pureza V, Walker PF. Chikungunya: Acute fever, rash and debilitating arthralgias in a returning traveler from Haiti. Journal of Travel Medicine. 2014; 21 (6): 418-420.  https://www.embase.com/search/results?subaction=viewrecord&id=L611880392&from=export U2 - L611880392 | The article is a case report. Design different from that established in the inclusion criterion: observational studies (case-control, cohort studies, cross-sectional studies). |
| 5 | Bandyopadhyay B, Pramanik N, R, Mukherjee H, Mukherjee H, K Neogi DK, Hatk IA. Chikungunya in West Bengal, India. Tropical Doctor. January 2009; 39: 59–60. doi: 10.1258/td.2008.080077. PMID: 19211436. | The article is a research letter |
| 6 | Bandyopadhyay D, Ghosh SK. Mucocutaneous manifestations of Chikungunya fever. Indian J Dermatol. 2010;55(1):64-7. doi: 10.4103/0019-5154.60356. PMID: 20418982; PMCID: PMC2856378. | The article is a review. Design different from that established in the inclusion criterion: observational studies (case-control, cohort studies, cross-sectional studies). |
| 7 | Barros AM, Martins-de-Barros AV, Costa MJ, Sette-de-Souza PH, Lucena EE, Araújo FA. Prevalence of ulcerative stomatitis in arbovirus infections in a Brazilian Northeast population. Medicina oral, patologia oral y cirugia bucal. 2020 ; 25 (6): pp. e810-e817 | The article did not discriminate the number of cases of oral manifestations only in chikungunya patients |
| 8 | Chandra P, Das B, Sowmya MK, Thimmaraju KV. Epidemiologal study on chikungunya outbreak in Mangalore district, Karnataka. Intl. Pharma Bio Sci. 2011; 2 (4): 342-7. doi: 10.4317/medoral.23865. PMID: 33037807; PMCID: PMC7648917. | Not getting the full text even after attempting to contact the authors |
| 9 | Chow A, Lye DC, Lee V, Win MK, Tan A, Leo YS. How different is chikungunya from dengue? Int. J. Infect. Dis.2010; 14: e371. doi:https://doi.org/10.1016/j.ijid.2010.02.446 | The article is a scientific abstracts from Congress proceedings of the 14th International Congress on Infectious Diseases (ICID) |
| 10 | Eber A, Perper M, Verne S, Magno R, Nouri K. Cutaneous manifestations of mosquito-borne illnesses in the Sunshine State. J. Am. Acad. Dermatol. 2017; 76 (6): pp. AB90.  https://www.embase.com/search/results?subaction=viewrecord&id=L617008801&from=export U2 - L617008801 | The article is an info article |
| 11 | De Figueiredo LQ, De Figueiredo ECQ, De Figueiredo JCQ. Sjögren's syndrome after chikungunya: case report. Advances in Rheumatology. 2008; 58.  https://www.embase.com/search/results?subaction=viewrecord&id=L624570671&from=export U2 - L624570671 | The article is a case report. Design different from that established in the inclusion criterion: observational studies (case-control, cohort studies, cross-sectional studies). |
| 12 | Del Castillo-Cabrera, S. Mucocutaneous manifestations of the chikunyunga fever. Dermatol Peru. 2014; 24 (3): 159-167. | The article is a review. Design different from that established in the inclusion criterion: observational studies (case-control, cohort studies, cross-sectional studies). |
| 13 | Ezzedin, K, Pistone T, Boisvert M, Receiver MC, Schuffenecker I, Zeller H, et al. Cluster of chikungunya virus in travelers returning from Senegal, 2006. J Travel Med. 2009; 16 (4): 286-8.  https://www.embase.com/search/results?subaction=viewrecord&id=L355073689&from=export U2 - L355073689 | The article is a case series. Design different from that established in the inclusion criterion: observational studies (case-control, cohort studies, cross-sectional studies). |
| 14 | Garg T, Sanke S, Ahmed R, Chander R, Basu S. Stevens-Johnson syndrome and necrolysis-like epidermal toxic cutaneous presentation of chikungunya fever: A case series. Pediatric Dermatology. 2018; 35 (3): 392-6.  doi: 10.1111/pde.13450. Epub 2018 Mar 24. PMID: 29573443 | The article is a case series. Design different from that established in the inclusion criterion: observational studies (case-control, cohort studies, cross-sectional studies). |
| 15 | Gueiros LA, Neves MCS, Marques CDL. Chikungunya fever and COVID-19: Oral ulcers are a common feature. Oral Dis. 2020 Nov 11. doi: 10.1111/odi.13717. Epub ahead of print. PMID: 33179342.. | The article is a case report. Design different from that established in the inclusion criterion: observational studies (case-control, cohort studies, cross-sectional studies). |
| 16 | Gunathilakel HMDR, Sirimanna GMP. Dermatological manifestations of Chikungunya fever. Sri Lanka Journal of Dermatology. 2006; 10: 52-3. https://www.slcd.lk/publications/articles/article/dermatological-manifestations-of-chikungunya-fever/ | The article is a case series. Design different from that established in the inclusion criterion: observational studies (case-control, cohort studies, cross-sectional studies). |
| 17 | Handler MZ, Handler NS, Stephany MP, Handler GA, Schwartz RA. Chikungunya fever: an emerging viral infection threatening North America and Europe. International journal of dermatology. 2007; 56 (2): pp. e19-e25.  https://doi.org/10.1111/ijd.13439 | The article is a review. Design different from that established in the inclusion criterion: observational studies (case-control, cohort studies, cross-sectional studies). |
| 18 | Hochedez P, Hausfater P, Jaureguiberry S, Gay F, Datry A, Danis M, et al. Cases of chikungunya fever imported from the islands of the South West Indian Ocean to Paris, France. Euro Surveill. 2007; 12 (1): 201–27.  https://doi.org/10.2807/esm.12.01.00679-en | The article is an outbreak report |
| 19 | Hyle EP, Alame D. Case 13-2015: A 27-Year-Old Woman with Arthralgias and a Rash. The new engl and journal of medicine illicit. 2015; 372 (17): 1657-64.  Hyle EP, Alame D. Case records of the Massachusetts General Hospital. Case 13-2015. A 27-year-old woman with arthralgias and a rash. N Engl J Med. 2015 Apr 23;372(17):1657-64. doi: 10.1056/NEJMcpc1415172. PMID: 25901430. | The article is a case report. Design different from that established in the inclusion criterion: observational studies (case-control, cohort studies, cross-sectional studies). |
| 20 | Kumar JC, Vivek Y, Sudhindra PK, Dhananjaya BD, Kumar AT, Guru K, et al. Oral candidiasis in Chikungunya viral fever: A case report. Cases Journal. 2010; 3: 6. doi: 10.1186/1757-1626-3-6. PMID: 20205786; PMCID: PMC2823716. | The article is a case report. Design different from that established in the inclusion criterion: observational studies (case-control, cohort studies, cross-sectional studies). |
| 21 | Labeaud AD, Noel T, Jungkind D, Fields P, Widjaja S, Mitchell G, et al. SAT 1054 Chikungunya fever in the Caribbean: clinical findings from Grenada. American Journal of Tropical Medicine and Hygiene. 2015; 93 (4 Suppl): 208. https://www.embase.com/search/results?subaction=viewrecord&id=L613370786&from=export U2 - L613370786 | The article is a scientific Abstracts from Congress proceedings of American Society of Tropical Medicine and Hygiene 2015 (Annual Meeting) |
| 22 | Leao JC, Marques C, Duarte A, de Almeida OP, Porter S, Gueiros LA. Chikungunya fever: General and oral healthcare implications. Oral diseases. 2018; 24 (1): pp. 233-237. doi: 10.1111/odi.12777. PMID: 29480628. | The article is a review. Design different from that established in the inclusion criterion: observational studies (case-control, cohort studies, cross-sectional studies). |
| 23 | Mac Donald-Ottevanger MS, Gravenberch-Ramnandanlall CI, Zijlmans CW. Chikungunya bij kinderen [Chikungunya in children]. Ned Tijdschr Geneeskd. 2015;159:A8403. Dutch. PMID: 25690071. | The article is a case series. Design different from that established in the inclusion criterion: observational studies (case-control, cohort studies, cross-sectional studies). |
| 24 | Miah, M T Islam, M R Ayaz, K M Patwary, M A Begum, A Shanchay, M S, et al. Experience of Chikungunya in a Chikungunya Clinic at Tertiary Medical College Hospital in Bangladesh Journal Article Observational Study Bangladesh Mymensingh Med J. 2020 Apr;29(2):431-437. https://pesquisa.bvsalud.org/portal/resource/pt/mdl-32506101 | Not getting the full text even after attempting to contact the authors |
| 25 | Mobeen N. Oral manifestation of chikungunya and dengue fever. Journal of Pharmaceutical Sciences and Research. 2015; 7 (9): 769-71. https://www.embase.com/search/results?subaction=viewrecord&id=L605792148&from=export U2 - L605792148 | The article is a review. Design different from that established in the inclusion criterion: observational studies (case-control, cohort studies, cross-sectional studies). |
| 26 | Monson B, Peper SM, Smith CJ. Debilitating joint pain following exotic travel. Journal of General Internal Medicine. 2015; 30: S384. https://www.embase.com/search/results?subaction=viewrecord&id=L71878223&from=export U2 - L71878223 | The article is a case report. Design different from that established in the inclusion criterion: observational studies (case-control, cohort studies, cross-sectional studies). |
| 27 | Muñoz CM, Castillo JO, Salas D, Valderrama MA, Rangel CT, Vargas HP, et al. ~~Silva DC.~~ [Atypical mucocutaneous manifestations in neonates and infants with chikungunya fever in the municipalities of Cúcuta, Los Patios and Villa del Rosario, Norte de Santander, Colombia, 2014]. Biomedica. 2016 Sep 1;36(3):368-377. Spanish. doi: 10.7705/biomedica.v36i3.2760. PMID: 27869384. | The article is a case series. Design different from that established in the inclusion criterion: observational studies (case-control, cohort studies, cross-sectional studies). |
| 28 | Norman FF, Monk-Maillo B, Perez-Molina NOW, By Ory F, Franco L, et al. Lymphadenopathy in Patients with Chikungunya Virus Infection Imported from Hispaniola: Case Reports. Journal of Travel Medicine. 2015; 22 (4): 272-5. doi: 10.1111/jtm.12204 | The article is a case series. Design different from that established in the inclusion criterion: observational studies (case-control, cohort studies, cross-sectional studies). |
| 29 | Padbidri VS, Gnaneswar TT. Epidemiological investigations of epidemic chikungunya at Barsi, Maharashtra state, India. J. Hyg Epidemiol Immunol. 1979; 23 (4): 445-51. PMID: 575900. | Not getting the full text even after attempting to contact the authors |
| 30 | Pandey A, Prakash V, Pandey M. SAT0390 Clinical spectrum of epidemic-observational study from a tertiary referral center in central India. Annals of the Rheumatic Diseases Jun 2018, 77 (SUPPL 2) 1058-1059; doi: 10.1136/annrheumdis-2018-eular.1482 | The article is a scientific abstracts from Congress proceedings of the Rheumatic Diseases |
| 31 | Pandey A. Clinical Manifestations of Chikungunya Fever in Patients with Ankylosing Spondylitis. Indian Journal of Rheumatology. 2019; 13 (4): 287-8  https://www.embase.com/search/results?subaction=viewrecord&id=L625163464&from=export U2 - L625163464 | The article is a letter to editor |
| 32 | Pandey A, Prakash V, Pandey M. Spectrum of chikungunya fever epidemic in cases of ankylosing spondylitis - in the observational study from a tertiary referral center in central India. Intern Med. J. 2018; 48: 16.  https://www.embase.com/search/results?subaction=viewrecord&id=L621898689&from=export U2 - L621898689 | The article contains partial reports of the included study Pandey A. Clinical Manifestations of Chikungunya Fever in Patients with Ankylosing Spondylitis. Indian Journal of Rheumatology. 2019; 13 (4): 287-8. |
| 33 | Panigrahi A, Chakraborty S, Sil A. Chik sign in chikungunya fever. Infection. 2020 Jul 1. doi: 10.1007/s15010-020-01472-x. Epub ahead of print. PMID: 32613531. | The article is a case report. Design different from that established in the inclusion criterion: observational studies (case-control, cohort studies, cross-sectional studies). |
| 34 | Paquet C, Quatresous I, Solet JL, Sissoko D, Renault P, et al. Chikungunya outbreak in Reunion: epidemiology and surveillance, 2005 to early January 2006. Euro Surveillance. 2006; 11 (5). doi: 10.2807/esw.11.05.02891-en | The article did not discriminate the number of cases of gingival bleeding. We have done contact with the authors, but they reported not having the data in question. |
| 35 | Paul BJ, Geetha P, Shanu PM. Clinical profile and long-term sequelae of chikungunya fever - A 1.5 year follow up study. Int. J. Rheum. Dis. 2010; 13: 194. https://www.embase.com/search/results?subaction=viewrecord&id=L70198258&from=export U2 - L70198258 | The article contains partial reports of included study Paul BJ, Pannarkady G, Moni SP, Thachil EJ. Clinical profile and long-term sequelae of Chikungunya fever. Indian J Rheumatol. 2011; 6 (1 SUPPL.): 12–9. |
| 36 | Ritz N, Hufnagel M, Gérardin P. Chikungunya in Children. Pediatr Infect Dis J. 2015 Jul;34(7):789-91. doi: 10.1097/INF.0000000000000716. PMID: 26069950. | The article is a review. Design different from that established in the inclusion criterion: observational studies (case-control, cohort studies, cross-sectional studies). |
| 37 | Shaikh,SH, Khan S. A rare variant of Guillain-Barre syndrome after a chikungunya viral fever J. Neurol. Sci. 2019; 405: pp. 29. https://www.embase.com/search/results?subaction=viewrecord&id=L2004005658&from=export U2 - L2004005658 | The article is a case report. Design different from that established in the inclusion criterion: observational studies (case-control, cohort studies, cross-sectional studies). |
| 38 | Scully C, Samaranayake LP. Emerging and changing viral diseases in the new millennium. Oral Diseases. 2016 Apri; 22 (3): 171-9. doi: 10.1111/odi.12356. Epub 2015 Aug 6. PMID: 26179810; PMCID: PMC7167660. | The article is a review. Design different from that established in the inclusion criterion: observational studies (case-control, cohort studies, cross-sectional studies). |
| 39 | Sharma P, Kaur J, Thami GP. Cutaneous features of chikungunya: a case series from north India. Journal of Pakistan Association of Dermatologists. 2018; 28 (3): 315-9.  https://www.embase.com/search/results?subaction=viewrecord&id=L625830740&from=export U2 - L625830740 | The article is a case series. Design different from that established in the inclusion criterion: observational studies (case-control, cohort studies, cross-sectional studies). |
| 40 | Thiberville SD, Boisson V, Gaudart J, Simon F, Flahault A, De Lamballerie X. Chikungunya Fever: A Clinical and Virologic Investigation of Outpatients on Reunion Island, South-West Indian Ocean. PLoS Neglected Tropical Diseases. 2013; 7 (1): e2004. doi: 10.1371/journal.pntd.0002004. Epub 2013 Jan 17. PMID: 23350006; PMCID: PMC3547841. | The article is a randomized control trial. Design different from that established in the inclusion criterion: observational studies (case-control, cohort studies, cross-sectional studies). |
| 41 | Valamparampil JJ, Chirakkarot S, Letha S, Jayakumar C, Gopinathan KM. Clinical profile of Chikungunya in infants. Indian Journal of Pediatrics. 2009 Feb; 76 (2): 151-5. doi: 10.1007/s12098-009-0045-x. Epub 2009 Mar 28. PMID: 19330303. | The article discriminates no evidence in oral cavity. |
