# Supplementary material for: Oral manifestations in chikungunya patients: A systematic review
Source: PLoS Negl Trop Dis. 2021 Jun 10;15(6):e0009401. doi: 10.1371/journal.pntd.0009401 (PMC8191910; doi:10.1371/journal.pntd.0009401)
Supplement: S1 Fig — PRISMA, Preferred Reporting Items for Systematic Reviews and Meta-Analyses. (DOCX) [file pntd.0009401.s006.docx]

**S1_Figure. PRISMA flow diagram.**

## Eligibility

## Screening

Additional records identified through other sources (Reference Lists)

**(n = 11)**

Records identified through database searching
**(n = 795)**

Medline (412); The Cochrane Library (25); SCOPUS (62); BVS (86); EMBASE (206); CAPES (04).

## Identification

Records after duplicates removed
**140 duplicates (n =655)**

Records excluded
**(n =598)**

Records screened
**(n =655)**

Full-text articles excluded, with reasons
**(n = 41)**

28 - Wrong study design

7 – Wrong publication type

3 - Wrong outcome evalueted

3 - Not get the full text

Full-text articles assessed for eligibility
**(n =68)**

Studies included in qualitative synthesis
**(n = 27)**

Studies included in quantitative synthesis (meta-analysis)
**(n = 0)**

## Included
